# Cross-omics analysis reveals microbe–metabolism interactions characteristic of gingival enlargement associated with fixed orthodontic in adolescents

**DOI:** 10.1080/20002297.2025.2513739

**Published:** 2025-06-04

**Authors:** Yang Lv, Sisi Peng, Yali Liu, Hefeng Yang, Guiding Li, Yi Peng

**Affiliations:** a Department of Periodontology, Kunming Medical University School and Hospital of Stomatology, Kunming, China; b Department of Orthodontics, Kunming Medical University School and Hospital of Stomatology, Kunming, China; c Kunming Medical University School and Hospital of Stomatology, Yunnan Key Laboratory of Stomatology, Kunming, China

**Keywords:** Orthodontics, gingival overgrowth, microbiomes, metagenomics, metabolomics

## Abstract

**Objectives:**

To investigate the oral microbiome and metabolome longitudinal changes associated with orthodontic treatment-induced gingival enlargement (OT-GE).

**Methods:**

Twenty-six subjects were divided into case and control groups based on the gingival overgrowth index (GOi). The OT-GE group was divided into the no gingival enlargement (OT-GE0, *n* = 5) and persistent gingival enlargement (OT-GE1, *n* = 11). The control group included orthodontic treatment periodontal health (OT-GH, *n* = 5), and no orthodontic treatment periodontal health (NOT-GH, *n* = 5). Microbial composition and metabolites in saliva were investigated using cross-omics.

**Results:**

Longitudinal analysis linked orthodontic treatment-induced gingival enlargement to distinct oral microbiome and metabolome shifts. The OT-GE group showed significantly higher bleeding on probing (BOP), plaque scores (*p* < 0.001), probing depth, GOi, and ligature wire differences (*p* < 0.05) versus controls. Microbial diversity and species richness were elevated in OT-GE (*p* < 0.05), though no differences emerged between OT-GE0 and OT-GE1) subgroup (*p* > 0.05). Cross-omics identified specific periodontal pathogens and metabolites linked to gingival enlargement. Disrupted amino acid biosynthesis pathways, particularly citrulline metabolism, correlated with functional gene dysregulation and microbial imbalance. Aberrant citrulline intake appeared to drive dysbiosis, potentially contributing to gingival overgrowth.

**Conclusions:**

OT-GE pathogenesis involves functional gene-regulated metabolite metabolism influencing periodontal pathogens.

## Introduction

Gingivitis and gingival enlargement are typical periodontal issues linked with orthodontic treatment [1], particularly among young patients. It has been reported that the frequency of Orthodontic treatment-induced gingival enlargement (OT-GE) is found to occur in 48% of patients aged 10 to19 years [2]. However, the etiology and mechanism of OT-GE are still unclear.

Orthodontic treatment has an impact on oral hygiene by promoting plaque retention, which can contribute gingival inflammation and enamel demineralization [1,3]. It is widely accepted that due to orthodontic brackets, archwires and other orthodontic appliances that interfere with plaque removal [4–6] will affect the composition of the oral microbiota [7,8]. Plaque microorganisms and their metabolites cause an immune-inflammatory response, resulting in gingivitis and gingival enlargement [9,10]. At the same time, as the gingival tissue enlarges, the surface of the teeth becomes more difficult to clean, resulting in poor dental hygiene and increased irritation and bleeding [11]. The pseudo-pockets formed by the hyperplastic gingival tissue may provide an anaerobic habitat for the proliferation of periodontal pathogenic bacteria, directly altering the microecologyl of the subgingival plaque and drastically changing its flora composition [9]. Yet there are individual variation in the gingival inflammatory response to plaque biofilms [12]. The explanation could be influenced by the quality or quantity of the plaque biofilm [13], as well as the host’s immunological response to the attack.

OT-GE has been associated to the presence of periodontal pathogens, such as *Treponema denticola* and *Prevotella intermedia* [9]. *Candida* may also be related with OT-GE [14]. However, the current research on the relationship between oral microbiota and orthodontic treatment has major limitations. To begin, several studies have analyzed oral microorganisms using just cross-sectional descriptive studies [13,15–18] or 16S rRNA gene sequencing [4,7], which only reacts to species-level microbial composition but not to strain subspecies and functional gene distributions. Second, high-throughput sequencing can only evaluate the differential colonies and does not correlate with downstream genes or metabolites, making it difficult to study illness onset and progression mechanism. Third, OT-GE should be discussed alongside periodontal healthy patients, with the microbiome of subjects serving as a control.

The microbiome refers to all the genomes and genetic material of microbial communities in certain habitats, as well as the metabolites they produce [19]. Gingival sulcus fluid, saliva, and blood include biomarkers that represent inflammatory and immunological responses to periodontal lesions [20], making metabolomic analysis a valuable tool for determining etiological and diagnosis. As a result, a cross-omic methodology with an orthodontic gingival enlargement group before and after treatment is required to reduce bias in the study process. Finally, in terms of treatment, OT-GE patients should have nonsurgical periodontal treatment to eliminate plaque biofilm and other contributing variables [21]. Even with professional periodontal treatment and the patient’s own plaque control, not all cases of gingival enlargement will be resolved. Therefore, we hypothesize that the persistence of OT-GE may be related to ‘specific bacteria’ and their metabolites.

This is the first study to look into the causes of gingival enlargement using high-resolution analysis of the oral microbiome and metabolites. We investigated 42 samples using shotgun metagenomics and untargeted metabolomics before finding microbial and metabolite profiles linked with orthodontic gingival enlargement using combination analysis.

## Materials and methods

### Study population

**Figure 1.**
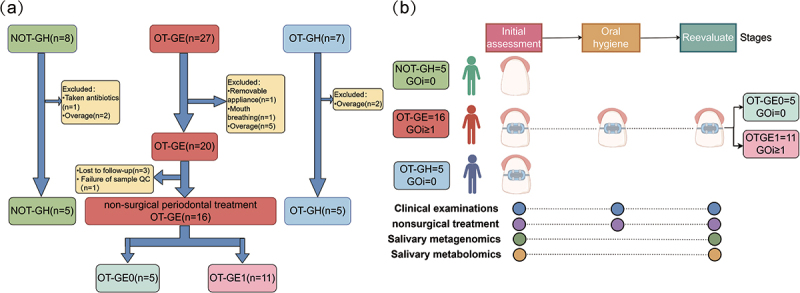
Subjects inclusion and clinical trial workflow. (a) Subgroups and screening for subjects inclusion and exclusion; (b) clinical trial design. NOT-GH, no orthodontic treatment group of periodontal health; OT-GH, orthodontic treatment group of periodontal health; OT-GE, group of orthodontic treatment-induced gingival enlargement; OT-GE0, group of no gingival enlargement after periodontal nonsurgical treatment; OT-GE1, group of gingival enlargement after periodontal nonsurgical treatment; GOi, gingival overgrowth index.

This study included 42 adolescents aged 10–19 years old who were treated and assessed at the Kunming Medical University Affiliated Stomatological Hospital in 2022–2023. The gingival overgrowth index (GOi) established by Angelopoulos & Goaz [22] and later refined by Miller & Damm [23] was used to measure vertically directed gingival enlargement using periodontal probe (PCP12/QOW6, Hu-Friedy). The case group was the OT-GE, with inclusion criteria of (1) fixed orthodontics; (2) gingivitis; and (3) GOi ≥ 1 mm. Following non-surgical periodontal treatment, the OT-GE group was separated into two subgroups: no gingival enlargement (OT-GE0, GOi = 0 mm) and persistent gingival enlargement (OT-GE1, GOi > 1 mm). The control group consisted of two groups: orthodontic treatment for periodontal health (OT-GH) and no orthodontic treatment for periodontal health (NOT-GH). Both control groups met the following criteria: (1) GOi = 0 mm; (2) Gingival health with no indication of periodontitis-related bone loss or attachment loss. The diagnostic criteria for both gingivitis and gingival health subjects in this study were in accordance with the consensus report of workgroup 1 of the 2017 world workshop on the classification of periodontal and peri-implant diseases and conditions [24]. Exclusion criteria for all groups: mouth breathing; having taken medications that may cause gingival hyperplasia, such as anticonvulsants, calcium channel blockers, and immunosuppressants; not having received systemic periodontal therapy and antibiotics within 6 months; smokers of any type and lost to visits. Detailed inclusion exclusions of cases and controls are shown in Figure 1(a).

### Study design and sample collection

In the initial assessment stage, we performed periodontal probing on all patients, recorded the periodontal chart (https://www.periodontalchart-online.com/uk/) and percentage of bleeding on probing (BOP%) [25], and after recording the GOi, all subjects were divided into OT-GE group, OT-GH group and NOT-GH group and saliva was collected. In addition, they received non-surgical periodontal treatment, including supragingival cleanings or subgingival scaling and root planning, and oral hygiene education. During the 2–4 week oral hygiene education phase, the OT-GE group was required to recall subjects for oral hygiene education to ensure that they were fully competent in tooth brushing and interdental brushing, and to examine the tooth surface at each visit using the Mira-2-Ton® (Hager & Werken, Duisburg, Germany) in order to visualise the extent of dental plaque. Plaque levels were scored using the Turesky Modified Quigley Hein plaque index (TQHPI) until the plaque score on the tooth surface was less than 2 [26]. 4 weeks later subjects would enter a reassessment phase in which patients in the case group were categorised into OT-GE0 and OT-GE1 on the base of the GOi (Figure 1(b)). All periodontal examinations were completed by two experienced periodontal specialists and standardized consistency tests were performed during the clinical assessment. Details of sample collection can be found in Supplementary Methods. After obtaining consent, orthodontist-related information was obtained from the orthodontist, including gender, age, bracket type, archwire type, ligature wire, orthodontic duration, and extraction correction.

### Bacterial shotgun metagenomics and non-target metabolomics analysis

Metagenomic sequencing was performed using a previously reported methodology consisting of three parts: DNA extraction, library preparation and data analysis [27]. Non-targeted metabolomics sequencing was consistent with that reported by Luo et al. [28]. After metabolite extraction, liquid chromatography mass spectrometry (LC-MS) detection, peak extraction and detection of down-loaded data, and finally data statistical analysis and visual mapping. Detailed metagenomic and non-targeted metabolomic experimental methods are described in Supplementary Methods.

### Association analysis of differential bacteria and differential metabolites

Association analysis will be performed for both metabolites and species, as well as metabolites and functional genes. Species correlation analysis investigates the association between microbial species structure and metabolites, whereas functional correlation analysis mostly use univariate correlation analysis to identify the link between differential metabolites and functional genes. Spearman’s correlation coefficients were determined between different species and metabolites, as well as between different metabolites and functional genes, in order to build the correlation matrix. The major strains found in metagenomics that were most related to grouping and environmental factors were then connected to metabolites and functional genes. Cytoscape [29] (version 3.10.1) was used for network visualisation. Only strong correlations (correlation coefficients greater than 0.65 or less than −0.65) were described in the combined differential species-differential metabolite-functional gene analysis.

### Statistical analysis

Statistical analyses were performed using R (Version 3.6.1; R Development Core Team) and SPSS (Version 22.0. Armonk, NY: IBM Corp). Different types of data are expressed using appropriate summary statistics. Means (standard deviations) were used as measurements that conformed to a normal distribution; measurements that did not conform to a normal distribution were expressed as medians (interquartile range). Fisher’s Exact Test were used to determine differences in baseline characteristics between cases and controls. One way ANOVA was used to determine the statistical significance of the normal data; Kruskal Wallis and two-sided Wilcoxon rank sum test were used to determine the statistical significance of the non-parametric data, and Benjamini-Hochberg False Discovery Rate (FDR) was used to correct for multiple hypotheses. Spearman’s correlation analysis was used to correlate parameters that were significant. In addition, receiver operating characteristic curve (ROC curves) were analysed for differential species using ‘MedCalc Statistical Software version 19.2.6 (MedCalc Software bv, Ostend, Belgium; https://www.medcalc.org; 2020)’ to screen for potential biomarkers. *p* value level < 0.05 was considered significant.

## Results

### Subject characteristics

Saliva samples were collected from a cross-sectional cohort of 26 subjects, including 16 gingival enlargement cases and 10 periodontal healthy controls, to investigate the relationship between oral microorganisms and their metabolites and gingival enlargement in fixed orthodontic adolescents. Gingival enlargement patients received oral hygiene education, and saliva samples were taken for shotgun metagenomics and untargeted metabolomics sequencing (Figure 1(b)).

Table 1 indicates that the BOP% were higher in the OT-GE group compared to the other groups (*p* < 0.05), and the differences in GOi scores and ligature wire types between the five groups were statistically significant (*p* < 0.05). They were all clinical parameters related to periodontics and orthodontics, with no statistically significant variations in gender, type of bracket, archwire type, orthodontic duration and tooth extraction.

**Table 1.** Initial assessment characteristics of the case and control groups.

Variables		NOT-GH^a^ (*n* = 5)	OT-GH^b^ (*n* = 5)	OT-GE^c^ (*n* = 16)	OT-GE0^d^ (*n* = 5)	OT-GE1^e^ (*n* = 11)	*P* value
Gender	male	3	2	6	3	3	0.677^†^
	female	2	3	10	2	8	
Probe depth	≤3 mm	5	5	0	5	0	<0.001^†^
	>3 mm	0	0	16	0	11	
BOP% ^f^ ///		6.8 ± 1.6*	7.8 ± 1.3*	51.4 ± 8.7	32.2 + 9.4*	29.0 ± 7.7*	<0.001^**‡**^
Goi^g^	GOi = 0	5	5	0	5	0	<0.001^†^
	GOi = 1	0	0	2	0	1	
	GOi = 2	0	0	3	0	6	
	GOi = 3	0	0	11	0	4	
TQHPI^h^	///	3.0(3.0,4.5)	5.0(3.5,5.0)	4.0(4.0,5.0)	1.0(1.0,1.0)*	1.0(1.0,1.0)*	<0.001^**§**^
Brackets type	Selfligating brackets	///	5	13	3	10	0.334^†^
	Conventional brackets	///	0	3	2	1	
Orthodontic arch type	Containing nickel	///	4	11	4	7	1.00^†^
	Steel	///	1	5	1	4	
Ligatures	Metal	///	1	10	5	5	0.031^†^
	Elastomerics	///	1	4	0	4	
	Both	///	0	2	0	2	
	Self-ligating	///	3	0	0	0	
Orthodontic duration (years)	<1	///	5	5	2	3	0.247^†^
	1–2	///	0	7	2	5	
	>2	///	0	4	1	3	
Extraction teeth	Nonextracted	///	3	4	1	3	0.585^†^
	Extracted	///	2	12	4	8	

†represents the corresponding clinical parameter R × C Fisher’s exact test; ‡ represents BOP% one-way ANOVA; § represents TQHPI’s Kruskal Walis test. * represents comparing with OT-GE group *p* < 0.05. ^a^No orthodontic treatment group of periodontal health, ^b^Orthodontic treatment group of periodontal health, ^c^Group of orthodontic treatment-induced gingival enlargement, ^d^Group of no gingival enlargement after periodontal nonsurgical treatment; ^e^Group of gingival enlargement after periodontal nonsurgical treatment, ^f^Percentage of bleeding on probing, ^g^Gingival overgrowth index, ^h^Turesky modified Quigley Hein plaque index.

### Taxonomic alterations associated with gingival enlargement identified by metagenomics

**Figure 2.**
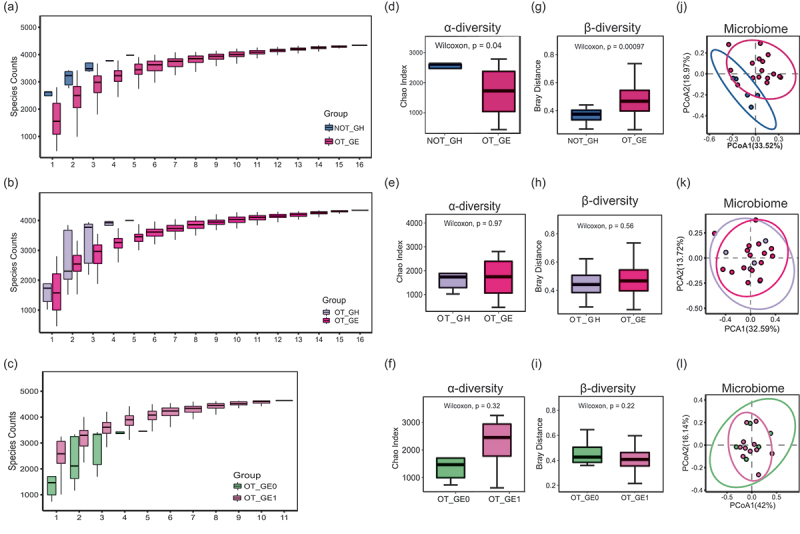
Oral microbial alterations in adolescents with gingival enlargement. (a-c) cumulative curves based on the number of species. The horizontal coordinate indicates the number of samples and the vertical coordinate indicates the number of species detected. (d-f) α-diversity (Chao index);(g-i), β-diversity (Bray-Curtis similarity index); (j-l) Principal Co-ordinates analysis (PCoA) based on species level. The confidence level of the elliptical confidence intervals of the scatterplot analyzed by PCoA is 95%. *P* < 0.05 indicates statistical significance. NOT-GH, no orthodontic treatment group of periodontal health; OT-GH, orthodontic treatment group of periodontal health; OT-GE, group of orthodontic treatment-induced gingival enlargement; OT-GE0, group of no gingival enlargement after periodontal nonsurgical treatment; OT-GE1, group of gingival enlargement after periodontal nonsurgical treatment.

To obtain adequate metagenomic sequencing depth and capture efficiency, the number of microbial bases in this study’s samples after de-hosting was all up to 20 Gb (Figure S1). As illustrated in Figures 2(a–c), the species accumulation curves plateaued with increasing sample size, suggesting sufficient sampling coverage to capture microbial diversity. Comparative analyses revealed that the OT-GE group exhibited significantly reduced α-diversity indices (*p* < 0.05) but markedly elevated *β*-diversity compared to the NOT-GH group (Figures 2(d,g); *p* < 0.01). Principal coordinates analysis (PCoA) further confirmed distinct clustering patterns between OT-GE and NOT-GH cohorts (Figure 2(j)), with greater dispersion in the OT-GE group indicating higher inter-sample heterogeneity. In contrast, no statistically significant differences in *α*-diversity (*p* > 0.05; Figures 2(e,f)) or *β*-diversity (*p* > 0.05; Figures 2(h–i)) were observed when comparing OT-GE to OT-GH, OT-GE0, or OT-GE1 subgroups. Consistently, PCoA ordination failed to demonstrate significant separation among these groups (Figures 2(k,l)), underscoring the specificity of microbial community shifts to persistent gingival enlargement.^1^^1^The indexes of richness and diversity of the microbiota in the case and control groups were shown in Figure S2.

Firmicutes, Proteobacteria, Actinobacteria, Bacteroidetes and Fusobacteria were the top five phyla in all groups (Figures S3a-c), which is consistent with a seminal work by Zaura [30] on the oral core microbiome. In the phylum level difference analysis, only NOT-GH and OT-GE had significantly different phyla. Proteobacteria more enriched in the NOT-GH group and negatively correlated with BOP and GOi, whereas Bacteroidetes and Spirochaetes were more enriched in the OT-GE group and positively correlated with BOP and GOi (Figures S3d-e, *p* < 0.05).

**Figure 3.**
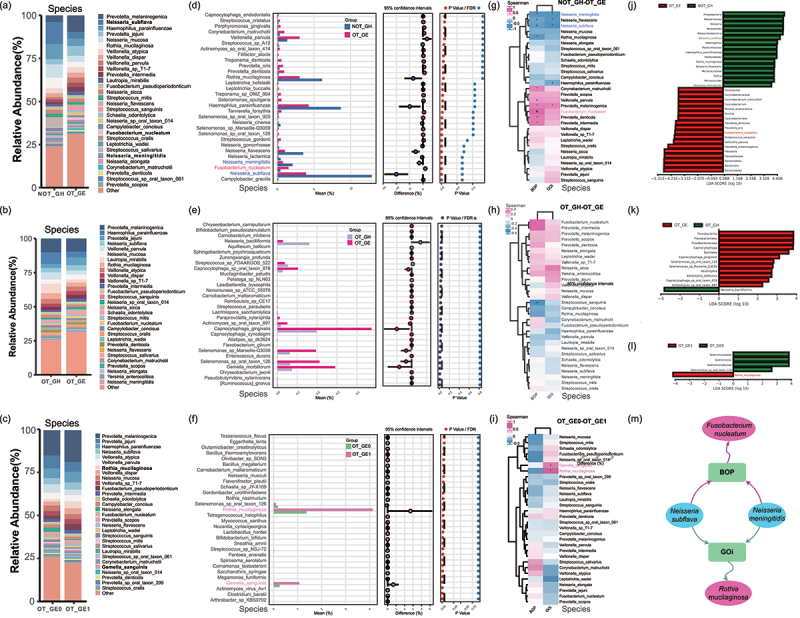
Associations of oral microorganisms with clinical parameters. (a-c) relative abundance of NOT-GH & OT-GE, OT-GH & OT-GE, and OT-GE0 & OT- GE1 at the bacterial species-level in the oral microbiota (Supplementary tables S1–3). (d-f) significant differences between microorganisms were identified by species-level system-theoretic accident model and processes (STAMP) analysis (Supplementary tables S16–18). (g-i) heat map of Spearman rank correlation coefficients between BOP, GOi, and species-level differential species (*P* < 0.05 marked ‘+’; *P* < 0.01 marked ’*’; *P* < 0.001 marked ‘#’; *P* > 0.05 not marked, Supplementary tables S25–27). (j-l) LEfSe analysis: the horizontal coordinate is the LDA value, the larger the LDA value indicates that it contributes more to the difference between the groups, the picture only shows the classification with LDA value greater than 2 (Supplementary tables S28–30). (m) Correlation networks between two clinical parameters and differential species. Red and green edges indicate correlation with BOP and correlation with GOi, respectively. The red ellipse indicates a significant positive correlation; the blue ellipse indicates a significant negative correlation.

More features were observed at the genus level, with *Prevotella*, *Veillonella*, *Neisseria*, *Streptococcus* and *Haemophilus* having the highest distributions in all groups (Figures S4a-c). Combined with clinical indicators, *Prevotella* and *Treponema* were positively correlated with OT-GE and BOP (*p* < 0.05), whereas *Rothia*, *Neisseria* and *Haemophilu* were significantly increased in NOT-GH (Figures S4d,g, *p* < 0.05). Notably analyzed in conjunction with the LEfSe LDA score, *Rothia* was nevertheless significantly associated with OT-GE in the NOT-GH versus OT-GE comparison group (Figure 3(j), *p* < 0.05). *Gemella* was positively correlated with OT-GE and GOi in OT-GH VS. OT-GE groups (Figures S4e,h, *p* < 0.05). *Selenomonas* was negatively correlated with OT-GE1 and GOi in the comparison group of OT-GE0 and OT-GE1 (Figures S4f,i) and was consistent with the LEfSe analysis (Figure 3(g)), suggesting that *Selenomonas* were associated with improvements in gingival enlargement.

### Association of oral microbial species with clinical indices

In the NOT-GH vs. OT-GE and OT-GH vs. OT-GE comparison groups, the top 30 species in terms of abundance share were demonstrated (Figures 3(a-c)); STAMP analysis presented the differential strains between the groups (Figures 3(d-f)); meanwhile, the BOP and GOi were used as the environmental factors, and Spearman’s corelation coefficients were computed for the relative abundance of the species and the environmental factors, and heatmaps were plotted (Figures 3(g-i)). LEfSe analysis was used to discover the species that best explained the between-group differences in the two sets of samples (Figures 3(j-l)). We found that no clinical parameter correlating strains could be found between the OT-GH vs. OT-GE groups, and thus could not be associated with strains that differed between the groups. For this reason, only NOT-GH & OT-GE comparison results will be shown subsequently in this study. Integrated analysis revealed a significant correlation between *Fusobacterium nucleatum* and BOP (*p* < 0.001), whereas *Neisseria meningitidis* and *Neisseria subflava* were negatively correlated with both BOP and GOi (*p* < 0.05, Figures 3(d,g)). Their ROC curves were analysed and the three key species had a combined AUC value of 0.963 (Figure S5), which can be used as a biomarker associated with OT-GE. Furthermore, in OT-GE0 vs. OT-GE1 comparison group, *Rothia mucilaginosa* and *Gemella sanguinis* were both significantly positively correlated with OT-GE1 and GOi, however, combined with the LEfSe LDA score, only *R. mucilaginosa* could be used as a biomarker (Figure 3(l)). Enrichment analysis of *R. mucilaginosa* and *G*. *sanguinis* showed that only *R*. *mucilaginosa* was statistically different, and ROC curve analysis was performed (Figure S6), with an AUC value of 0.855, suggesting that *R*. *mucilaginosa* can serve as a biomarker associated with OT-GE1. The above key species and clinical correlations were summarized in Figure 3(m).

### Functional characterization of metabolites associated with gingival enlargement

Only NOT-GH vs. OT-GE group and OT-GE0 vs. OT-GE1 group were able to correspond to the clinical parameter correlation strain (*p* < 0.05) with the between-group difference strain (*p* < 0.05). Therefore, in order to facilitate the subsequent association analysis, this part only shows the results of visualization analysis between NOT-GH group vs. OT-GE group and OT-GE0 group vs. OT-GE1 group with subsequent discussion.

Metabolite peaks were identified from raw mass spectrometry data and annotated with KEGG, HMDB, and public databases. In the NOT-GH vs. OT-GE comparison, 221 metabolites were up-regulated and 142 were down-regulated; in the OT-GE0 vs. OT-GE1 comparison, 21 metabolites were up-regulated and 67 were down-regulated (Figures S7a-b).

Differential metabolite rows were analyzed for KEGG database pathway enrichment and the top 10 metabolic pathways of p-value were plotted in bubble and network diagrams (Figures S7c-d and S8). We found that citrulline (L-Citrulline, Citrulline) was the co-existing differential metabolite in both NOT-GH vs. OT-GE and OT-GE0 vs. OT-GE1 comparator groups (Figures S7c-d) and was associated with the biosynthesis of amino acids (KEGG KO pathway map01230) and arginine biosynthesis (KEGG KO pathway map00220) pathways (Figure S8a). Furthermore, the amino acid biosynthesis pathway was the pathway that NOT-GH vs. OT-GE and OT-GE0 vs. OT-GE1 were co-enriched (Figure S8b). Further statistical analysis of the distribution of citrulline among the groups showed that citrulline was significantly under-expressed in the OT-GE group, but especially in the OT-GE1 group in which gingival enlargement had not yet receded after periodontal treatment (Figures S7e-f, *p* < 0.05).

### Integrated analysis of the oral microbiome, metabolome and differentially functional genes

**Figure 4.**
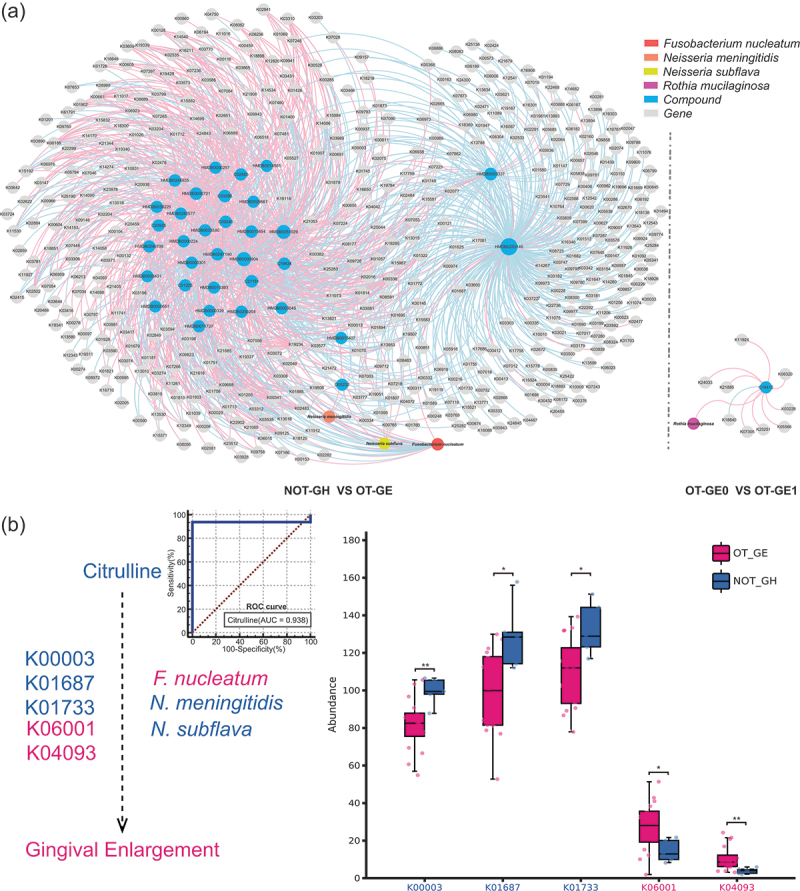
Co-occurrence analysis of potentially pathogenic species-metabolite-function genes associated with gingival enlargement. (a) Co-occurrence network was clustered according to species-metabolite-gene, species including *F. nucleatum*, *N. meningitidis*, *N. subflava* and *R. mucilaginosa* were the core of the network, and the association allowed us to identify the most critical metabolite and functional genes, which supports their role in disease progression. The size of the nodes represents the number of genes associated. Blue edge indicates Spearman rank correlation coefficient < −0.65, *P* < 0.05; red edge, Spearman rank correlation coefficient > 0.65, *p* < 0.05. Label of compounds and genes represents KEGG/HMDB numbers (Supplementary tables S31–38). (b) Left side, ROC curves and AUC values of screened citrulline. *F. nucleatum*, *N. meningitidis* and *N. subflava* have genes involved in citrulline metabolism in the control NOT-GH-enriched (blue) and gingival enlargement group OT-GE-enriched (red). The right panel shows the two-sided Wilcoxon rank sum test for KOs in NOT-GH and OT-GE. K00003 was identified as homoserine dehydrogenase; K04093, chorismate mutase; K06001, tryptophan synthase beta chain; K01687, dihydroxy-acid dehydratase; K01733, threonine synthase, and the enzyme reactions involved are shown in supplemental table 35. *p < 0.05; **p < 0.01.

The microorganisms associated with orthodontic gingival enlargement described above were analyzed in a co-occurrence network with differential metabolites and functional genes (Figure 4(a)). Species including *N*. *meningitidis*, *N. subflava* and *F*. *nucleatum* were at the core of this network, and their common metabolites and functional genes could be found by association, with compounds and genes labelled to represent KEGG or HMDB numbers.

Citrulline was the only chemical that showed enrichment with *F. nucleatum*, *N. meningitidis*, and *N. subflava* in the NOT-GH vs. OT-GE. Although the OT-GE0 vs. OT-GE1 comparison group was also enriched for the differential metabolite thiourea (KEGG ID C14415), which is associated with *R*. *mucilaginosa*, it was not enriched for any metabolic pathway. Citrulline thus stood out, and we followed the trail to locate the functional genes related to the metabolic pathways in which it resides: arginine biosynthesis (KEGG ID map00220) and biosynthesis of amino acids (KEGG ID map01230).

In order to verify whether these functional genes are under gene regulation by *N*. *meningitidis*, *N*. *subflava* and *F*. *nucleatum*, we compared the genome sequences of their standard strains in NBCI, and the results showed that five genes of amino acid biosynthesis pathway existed in their genomes at the same time. The above results suggest that *N*. *meningitidis*, *N*. *subflava* and *F. nucleatum* may be involved in the process of citrulline metabolism in some way. Their relationship is shown in Figure 4(b) left. Figure 4(b) right shows the distribution of five key functional genes in NOT-GH and OT-GE. The functional gene IDs and enzyme reactions involved are shown in Supplementary Table S39.

## Discussion

Despite significant advances in microbiome research [4–6] during orthodontic treatment, few studies have shown a relationship between gingival overgrowth and microbiota. In this investigation, DNBSEQ-T7 platform-based metagenomics combined with MS-based metabolomics was used to study the phenotypic changes between OT-GE adolescents and healthy controls. Association analysis was used to screen the core flora associated with OT-GE and its metabolites and potential functional genes.

Gingival overgrowth caused by orthodontic therapy might cover the tooth surface, putting oral hygiene and cleanliness at risk. This can cause an increase in bacterial burden, which may last even after orthodontic appliances are removed [31]. Thus, in clinical practice, surgical therapy is frequently indicated in instances where non-surgical interventions fail to improve function and appearance. In addition, the type of orthodontic therapy influences the prognosis. A new study discovered that fixed orthodontic treatment affects the plaque community, resulting in poorer oral health than clear aligners [1]. Daniel [32] found that the salivary microbiota reflects local bacterial changes in the supragingival and subgingival microbiota. Thus, the current study examined the salivary microbiota cross-omic analysis of the occurrence of OT-GE in teenagers.

Certain nuances were indicated by relative abundance at the phylum level: the OT-GE group had a significant rise in Bacteroidetes and Spirochaetes (Figure S3d). Bacteroidetes is a phylum found throughout the gut and mouth microbiome. Notably, Kado observed a rise in the Bacteroidetes phylum as orthodontic therapy continued [7]. Spirochaetes account for 50% of the polymicrobial flora in subgingival plaque in people with periodontitis, but less than 1% in healthy populations [33]. However, the individuals in this study had gingivitis and had the similar pattern as periodontitis.

*Prevotella*, a dominant genus of Bacteroidetes, is a specialist anaerobic bacteria closely associated with periodontal infections [34]. It was significantly increased in OT-GE and significantly positively correlated with BOP and GOi (Figures S4d,g), consistent with Tana’s report that *Prevotella* is associated with gingivitis after orthodontics treatment [35]. Although Gemalla is enriched in the OT-GE group and associated with GOi (Figures S4e,h), previous studies have suggested that it is more likely to be present in healthy oral ecology [36]. Furthermore, *Rothia*, *Neisseria*, and *Haemophilus* were considerably reduced in OT-GE and significantly increased in NOT-GH. Several recent studies linking *Rothia*, *Neisseria*, and *Haemophilus* to dental health [37–39] are in agreement with our results. *Selenomonas* was reduced in OT-GE1 and increased in NOT-GH, and they both were negatively correlated with BOP or GOi (Figure S4). *Selenomonas* spp. are important in periodontitis plaque biofilms and are thought to be related to the periodontal pathogen *F. nucleatum* [40], but Drescher’s report [41] supports our results that *Selenomonas* are insufficient biomarkers to diagnose periodontal disease.

Investigation at the species level yielded interesting results. In comparing NOT-GH vs. OT-GE (Figure 3(d)), we found that certain anaerobic pathogens of periodontitis, such as *Tannerella forsythia* (*p* < 0.05), *T. denticola* (*p* < 0.05), *Porphyromonas gingivalis* (*p* < 0.01) were considerably enriched in OT-GE. A study on the effect of fixed orthodontic appliances on the oral microbiota discovered that orthodontic appliances can cause the oral microbiota to shift from healthy to periodontitis [7]. Although the OT-GE group in this study had only gingivitis, periodontitis-associated microorganisms may indeed increase the likelihood of inflammation. Previous longitudinal clinical investigations have demonstrated that gingivitis is a risk factor for periodontitis [42,43], but it is unclear whether and how the gingivitis-associated microbiota contributes to periodontitis.

Our results (Figure 3) showed a large increase in *F*. *nucleatum* in OT-GE (positively connected with BOP) and a significant decrease in *N*. *meningitidis* and *N*. *subflava* (both negatively correlated with BOP and GOi). *F*. *nucleatu*m is a gram-negative, anaerobic bacterium that is thought to be the most prevalent in the oral cavity. They can either operate as commensal bacteria in the oral environment, connecting early and late colonizing bacteria and encouraging the growth and maturation of plaque biofilms [44,45], or they can be engaged in oral diseases [46–49]. *F. nucleatum* triggers immunological inflammation by increasing the production of *β*-defensin 2 and pro-inflammatory cytokines in the oral epithelium [50–52]. This could explain why *F*. *nucleatum* was linked to bleeding on probing in our study, as BOP is a direct result of periodontal inflammation. Although *N*. *meningitidis* is a known pathogen, *Neisseria* spp. are commonly believed to be part of the normal bacterial ecology of the oral cavity [53], and some studies have found a link between *Neisseria* spp. and improved oral health or reduced gingivitis [35,54]. E. Babikow discovered a significant link between *N*. *subflav*a and lower BOP [55], and a research of the oral microbiome in periodontally healthy people found a correlation between *N*. *subflava* and periodontal health [56]. Meanwhile, we highlighted a prior work that found that *N*. *meningitidis* and *N*. *subflava* are allelic, and that *N*. *meningitidis* can acquire drug resistance from *N*. *subflava* via horizontal gene transfer [57]. This shows that *N. meningitidis* may need to adapt to its host’s ecological niche to function. In short, our findings imply that *N. meningitidis* and *N. subflava* are associated with lower BOP and GOi.

In contrast, *R*. *mucilaginosa* and *Gemella sanguini*s were associated with persistent gingival enlargement when OT-GE0 was compared to OT-GE1 (Figures 3(f,i)). A prior investigation found a link between *G*. *sanguinis* and gingivitis [35], which is congruent with our results. When paired with LDA analysis (Figure 3(h)) and ROC analysis (Figure S6), we believe *R*. *mucilaginosa* is a more persuasive strain related with persistent gingival enlargement (OT-GE1). *R*. *mucilaginosa* is a well-studied *Rothia* species that is frequently found in the oral cavity. A caries-related microbiota investigation discovered that *Rothia* spp., particularly *R*. *mucilaginosa*, were more prevalent in the saliva of healthy children than in the saliva of carious children [37]. In another study of the oral squamous cell carcinoma microbiome, *R*. *mucilaginosa* was discovered to be more numerous in non-cancerous controls [58]. In contrast, earlier research has connected *R*. *mucilaginosa* to caries [59,60]. This heterogeneity of *R*. *mucilaginosa* in the disease was also seen in our analysis, as evidenced by enrichment in the NOT-GH and OT-GE1 groups (Figures 3(d,f)). We speculated that this was due to the involvement of specific *R*. *mucilaginosa* metabolites in the pathogenesis of chronic gingival enlargement, but no statistically significant link was discovered in our experiment.

Additional excavation provided strong support for the preceding conclusions. Citruline was a significant differential metabolite across the comparison groups (Figure S7). Citrulline, a non-essential amino acid, aids in the maintenance of normal levels of nitric oxide (NO), which has a role in the etiology of periodontal disease [61]. It is produced by *P*. *gingivalis*, the only bacterium with the enzyme arginine deaminase (PAD), which converts arginine to citrulline. Previous research has consistently connected citrulline to periodontitis [62,63]. Citrulline was significantly more prevalent in OT-GE1 than OT-GE0 in the current investigation (Figure S7f), indicating a relationship to long-term gingival overgrowth. In contrast, the OT-GE group had lower citrulline levels than the NOT-GH group (Figure S7e). Meanwhile, citrulline was negatively correlated with *F*. *nucleatum* but positively correlated with *N*. *meningitidis* and *N*. *subflava* (Figure 4(a), |coefficients| >0.65, *p* < 0.05). We hypothesize that low citrulline enrichment encourages *F*. *nucleatum* to accumulate in the oral environment while reducing *N*. *meningitidis* and *N*. *subflava*. Increased levels of pathogenic *F*. *nucleatum* may raise the risk of BOP and inflammation.

**Figure 5.**
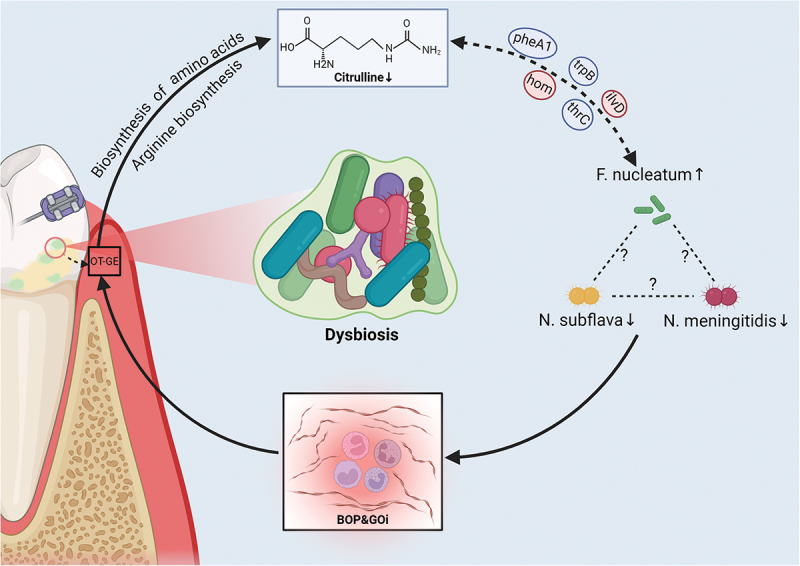
Potential mechanisms by which disorders of citrulline metabolism induce dysbiosis leading to OT-GE development.

Note: hom, homoserine dehydrogenase; pheA1, chorismate mutase; trpB, tryptophan synthase beta chain; ilvD, dihydroxy-acid dehydratase; thrC, threonine synthase; BOP, Bleeding on probing; GOi, gingival overgrowth index. OT-GE, orthodontic treatment-induced gingival enlargement.

Therefore, the above results suggest that dysbiosis due to citrulline depletion may be one of the causes of gingival hyperplasia. Since citrulline expression is regulated by functional genes of the amino acid metabolism pathway, a certain state after non-surgical periodontal treatment may lead to the enrichment of citrulline in periodontal tissues, and abnormalities in the metabolic level of such amino acids may be the cause of persistent gingival hyperplasia after non-surgical periodontal treatment. Oral microecology is a crucial system in the human body that comprises mostly of the oral microbiota and the host’s intrinsic oral environment. Oral microorganisms improve oral health by actively interacting with the host. When the balance is broken, oral infectious diseases can develop [64]. More than one bacterium appears to be causing periodontal disease [65]; infectious symbionts integrating [66], trading metabolites [67], and coexisting with the host may be to blame for the imbalance in the oral environment’s homeostasis. In this study, citrulline deficit was connected with an increase in *F*. *nucleatu*m, which was linked to BOP. Proteomic analysis demonstrated that the metabolic pathways of *F*. *nucleatum* include amino acid fermentation and glycolysis [68], implying that *F*. *nucleatum* may be impacted by other microbes in specific way. Figure 5 depicts one possible method by which citrulline metabolism abnormalities cause dysbiosis, resulting in the formation of OT-GE. We hypothesize that the three strains selected play a role in the development of OT-GE and may be useful targets for illness detection and treatment (Figure S6).

The cross-sectional design was the main limitation of this study, but we also analyzed the longitudinal microbiota before and after non-surgical treatment of the gingival enlargement group, and we attempted to explain the response of the microbial community and its metabolites as the treatment progressed, focusing on analyses of (1) differences between groups as well as between groups and time, and (2) network modeling, which allows the capture of complex microbial interactions. This may provide guidelines for future study to advance OT-GE. Another disadvantage of this study is the tiny sample size for a single group. Although we began the investigation with a greater number of patients, we did subject exclusion based on age and COVID-19 antibiotic use to reduce bias caused by various types of patients. We attempted to probe deeper into the available samples by increasing the depth of sequencing during shotgun metagenomics and metabolomics experiments. Furthermore, the effect of orthodontics on periodontal health varies by age [2,69]. The study population for the current study was adolescents aged 10 to 19, who have a higher prevalence; however, there may be some limitations. Adolescent plaque microorganisms differ from those of other age groups [70]; and during puberty, higher hormone levels (such as progesterone and possibly oestrogen) might cause increased blood flow to the gums [71]. This may increase the gums’ sensitivity, resulting in a higher reaction to any stimuli, including food and plaque.

## Conclusion

This high-resolution study of metabolites and the oral microbiome provides valuable insights for future research. *F*. *nucleatum*, *N*. *meningitidis*, and *N*. *subflava* have all been identified as useful indications of OT-GE-related dysbiosis. This will provide a fresh approach to diagnosing gingival enlargement. Our understanding of how orthodontics impacts oral microorganisms will be enhanced by continued research into the species of oral bacteria associated with orthodontic treatment, as well as the mechanisms behind their upstream genes and downstream metabolites. This could lead to the development of innovative medicines like probiotics and targeted antibiotics.

## Supplementary Material

Supplementary figures and methods.pdf

## Data Availability

Supplementary tables can be found in the online version of this article (http://wailianq.w2060.cn/Supplementary%20Tables%20andText.xlsx). The raw sequence data reported in this paper have been deposited in the Genome Sequence Archive in National Genomics Data Center, China National Center for Bioinformation, Chinese Academy of Sciences (Metagenome GSA: CRA041186, OMIX database: OMIX010010) that are publicly accessible at https://ngdc.cncb.ac.cn/gsa.

## References

[1] Shokeen B, Viloria E, Duong E, et al. The impact of fixed orthodontic appliances and clear aligners on the oral microbiome and the association with clinical parameters: a longitudinal comparative study. Am J Orthod Dentofacial Orthop. 2022;161(5):e475–14. doi: 10.1016/j.ajodo.2021.10.015 35248417

[2] Eid HA, Assiri HA, Kandyala R, et al. Gingival enlargement in different age groups during fixed orthodontic treatment. J Int Oral Health. 2014;6(1):1–4. PMC3959129 24653595

[3] Bergamo AZN, Nelson-Filho P, Do Nascimento C, et al. Cytokine profile changes in gingival crevicular fluid after placement different brackets types. Arch Oral Biol. 2018;85:79–83. doi: 10.1016/j.archoralbio.2017.09.022 29032048

[4] Marincak Vrankova Z, Rousi M, Cvanova M, et al. Effect of fixed orthodontic appliances on gingival status and oral microbiota: a pilot study. BMC Oral Health. 2022;22(1):455. doi: 10.1186/s12903-022-02511-9 36303145 PMC9615380

[5] van Gastel J, Quirynen M, Teughels W, et al. Longitudinal changes in microbiology and clinical periodontal variables after placement of fixed orthodontic appliances. J Periodontol. 2008;79(11):2078–2086. doi: 10.1902/jop.2008.080153 18980516

[6] van Gastel J, Quirynen M, Teughels W, et al. Longitudinal changes in microbiology and clinical periodontal parameters after removal of fixed orthodontic appliances. Eur J Orthod. 2011;33(1):15–21. doi: 10.1093/ejo/cjq032 20671070

[7] Kado I, Hisatsune J, Tsuruda K, et al. The impact of fixed orthodontic appliances on oral microbiome dynamics in Japanese patients. Sci Rep. 2020;10(1):21989. doi: 10.1038/s41598-020-78971-2 33319834 PMC7738506

[8] Bergamo AZN, Casarin RCV, Do Nascimento C, et al. Self-ligating brackets exhibit accumulation of high levels of periodontopathogens in gingival crevicular fluid. Odontology. 2022;110(3):460–466. doi: 10.1007/s10266-021-00677-2 35037112

[9] Gong Y, Lu J, Ding X. Clinical, microbiologic, and immunologic factors of orthodontic treatment-induced gingival enlargement. Am J Orthod Dentofacial Orthop. 2011;140(1):58–64. doi: 10.1016/j.ajodo.2010.02.033 21724088

[10] Sioustis IA, Martu MA, Aminov L, et al. Salivary metalloproteinase-8 and metalloproteinase-9 evaluation in patients undergoing fixed orthodontic treatment before and after periodontal therapy. Int J Environ Res Public Health. 2021;18(4):18. doi: 10.3390/ijerph18041583 PMC7915089 33567492

[11] Manuelli M, Marcolina M, Nardi N, et al. Oral mucosal complications in orthodontic treatment. Minerva Stomatol. 2019;68(2):84–88. doi: 10.23736/S0026-4970.18.04127-4 30854838

[12] Trombelli L, Farina R. A review of factors influencing the incidence and severity of plaque-induced gingivitis. Minerva Stomatol. 2013;62(6):207–234. 23828258

[13] Vincent-Bugnas S, Borsa L, Gruss A, et al. Prioritization of predisposing factors of gingival hyperplasia during orthodontic treatment: the role of amount of biofilm. BMC Oral Health. 2021;21(1):84. doi: 10.1186/s12903-021-01433-2 33627113 PMC7903590

[14] Tapia CV, Batarce C, Amaro J, et al. Microbiological characterisation of the colonisation by Candida sp in patients with orthodontic fixed appliances and evaluation of host responses in saliva. Mycoses. 2019;62(3):247–251. doi: 10.1111/myc.12880 30561858

[15] Almansob YA, Alhammadi MS, Luo XJ, et al. Comprehensive evaluation of factors that induce gingival enlargement during orthodontic treatment: a cross-sectional comparative study. Niger J Clin Pract. 2021;24(11):1649–1655. doi: 10.4103/njcp.njcp_69_21 34782504

[16] Pinto AS, Alves LS, Zenkner J, et al. Gingival enlargement in orthodontic patients: effect of treatment duration. Am J Orthod Dentofacial Orthop. 2017;152(4):477–482. doi: 10.1016/j.ajodo.2016.10.042 28962731

[17] Zanatta FB, Ardenghi TM, Antoniazzi RP, et al. Association between gingivitis and anterior gingival enlargement in subjects undergoing fixed orthodontic treatment. Dental Press J Orthod. 2014;19(3):59–66. doi: 10.1590/2176-9451.19.3.059-066.oar 25162567 PMC4296628

[18] Zanatta FB, Ardenghi TM, Antoniazzi RP, et al. Association between gingival bleeding and gingival enlargement and oral health-related quality of life (OHRQoL) of subjects under fixed orthodontic treatment: a cross-sectional study. BMC Oral Health. 2012;12(1):53. doi: 10.1186/1472-6831-12-53 23186371 PMC3534331

[19] Berg G, Rybakova D, Fischer D, et al. Microbiome definition re-visited: old concepts and new challenges. Microbiome. 2020;8(1):103. doi: 10.1186/s40168-020-00875-0 32605663 PMC7329523

[20] Lu H, Zou P, Zhang Y, et al. The sampling strategy of oral microbiome. iMeta. 2022;1(2):e23. doi: 10.1002/imt2.23 38868567 PMC10989882

[21] Newman MG, Takei HH, Klokkevold PR, et al. Newman and Carranza’s clinical periodontology. Elsevier; 2019.

[22] Angelopoulos AP, Goaz PW. Incidence of diphenylhydantoin gingival hyperplasia. Oral Surg Oral Med Oral Pathol. 1972;34(6):898–906. doi: 10.1016/0030-4220(72)90228-9 4509004

[23] Miller CS, Damm DD. Incidence of verapamil-induced gingival hyperplasia in a dental population. J Periodontol. 1992;63(5):453–456. doi: 10.1902/jop.1992.63.5.453 1527689

[24] Chapple ILC, Mealey BL, Van Dyke TE, et al. Periodontal health and gingival diseases and conditions on an intact and a reduced periodontium: consensus report of workgroup 1 of the 2017 world workshop on the classification of periodontal and peri-implant diseases and conditions. J Periodontol. 2018;89 Suppl 1(Suppl 1):S74–s84. doi: 10.1002/JPER.17-0719 29926944

[25] Ainamo J, Bay I. Problems and proposals for recording gingivitis and plaque. Int Dent J. 1975;25:229–235. 1058834

[26] Turesky S, Gilmore ND, Glickman I. Reduced plaque formation by the chloromethyl analogue of victamine C. J Periodontol. 1970;41(41):41–43. doi: 10.1902/jop.1970.41.41.41 5264376

[27] Jie Z, Chen C, Hao L, et al. Life history recorded in the Vagino-cervical microbiome along with multi-omes. Genomics Proteomics Bioinf. 2022;20(2):304–321. doi: 10.1016/j.gpb.2021.01.005 PMC9684086 34118463

[28] Luo S, Lou F, Yan L, et al. Comprehensive analysis of the oral microbiota and metabolome change in patients of burning mouth syndrome with psychiatric symptoms. J Oral Microbiol. 2024;16(1):2362313. doi: 10.1080/20002297.2024.2362313 38835338 PMC11149574

[29] Shannon P, Markiel A, Ozier O, et al. Cytoscape: a software environment for integrated models of biomolecular interaction networks. Genome Res. 2003;13(11):2498–2504. doi: 10.1101/gr.1239303 14597658 PMC403769

[30] Zaura E, Keijser BJ, Huse SM, et al. Defining the healthy “core microbiome” of oral microbial communities. BMC Microbiol. 2009;9(1):259. doi: 10.1186/1471-2180-9-259 20003481 PMC2805672

[31] Kouraki E, Bissada NF, Palomo JM, et al. Gingival enlargement and resolution during and after orthodontic treatment. New Y State Dent J. 2005;71(4):34–37. 16146305

[32] Belstrøm D. The salivary microbiota in health and disease. J Oral Microbiol. 2020;12(1):1723975. doi: 10.1080/20002297.2020.1723975 32128039 PMC7034443

[33] Chan EC, McLaughlin R. Taxonomy and virulence of oral spirochetes. Oral Microbiol Immunol. 2000;15(1):1–9. doi: 10.1034/j.1399-302x.2000.150101.x 11155157

[34] Nadkarni MA, Browne GV, Chhour KL, et al. Pattern of distribution of prevotella species/phylotypes associated with healthy gingiva and periodontal disease. Eur J Clin Microbiol Infect Dis. 2012;31(11):2989–2999. doi: 10.1007/s10096-012-1651-5 22684253

[35] Tanner AC, Sonis AL, Lif Holgerson P, et al. White-spot lesions and gingivitis microbiotas in orthodontic patients. J Dent Res. 2012;91(9):853–858. doi: 10.1177/0022034512455031 22837552 PMC3420397

[36] Torres-Morales J, Mark Welch JL, Dewhirst FE, et al. Site-specialization of human oral gemella species. J Oral Microbiol. 2023;15(1):2225261. doi: 10.1080/20002297.2023.2225261 37361319 PMC10288933

[37] Baker JL, Morton JT, Dinis M, et al. Deep metagenomics examines the oral microbiome during dental caries, revealing novel taxa and co-occurrences with host molecules. Genome Res. 2021;31(1):64–74. doi: 10.1101/gr.265645.120 33239396 PMC7849383

[38] Havsed K, Stensson M, Jansson H, et al. Bacterial composition and metabolomics of dental plaque from adolescents. Front Cell Infect Microbiol. 2021;11:716493. doi: 10.3389/fcimb.2021.716493 34395316 PMC8362896

[39] Agnello M, Marques J, Cen L, et al. Microbiome associated with severe caries in Canadian first Nations children. J Dent Res. 2017;96(12):1378–1385. doi: 10.1177/0022034517718819 28709393 PMC5652857

[40] Hawkes CG, Hinson AN, Vashishta A, et al. Selenomonas sputigena interactions with gingival epithelial cells that promote inflammation. Infect Immun. 2023;91(2):e0031922. doi: 10.1128/iai.00319-22 36648232 PMC9933688

[41] Drescher J, Schlafer S, Schaudinn C, et al. Molecular epidemiology and spatial distribution of selenomonas spp. In subgingival biofilms. Eur J Oral Sci. 2010;118(5):466–474. doi: 10.1111/j.1600-0722.2010.00765.x 20831580

[42] Tanner AC, Kent R, Kanasi E, et al. Clinical characteristics and microbiota of progressing slight chronic periodontitis in adults. J Clin Periodontol. 2007;34(11):917–930. doi: 10.1111/j.1600-051X.2007.01126.x 17877747

[43] Schätzle M, Löe H, Bürgin W, et al. Clinical course of chronic periodontitis. I. Role of gingivitis. J Clin Periodontol. 2003;30(10):887–901. doi: 10.1034/j.1600-051X.2003.00414.x 14710769

[44] Mark Welch JL, Rossetti BJ, Rieken CW, et al. Biogeography of a human oral microbiome at the micron scale. In: *Proceedings of the National Academy of Sciences of the United States of America*; 2016. p. E791–800. 10.1073/pnas.1522149113 PMC4760785 26811460

[45] Brennan CA, Garrett WS. Fusobacterium nucleatum — symbiont, opportunist and oncobacterium. Nat Rev Microbiol. 2019;17(3):156–166. doi: 10.1038/s41579-018-0129-6 30546113 PMC6589823

[46] Arenas Rodrigues VA, de Avila ED, Nakano V, et al. Qualitative, quantitative and genotypic evaluation of aggregatibacter actinomycetemcomitans and fusobacterium nucleatum isolated from individuals with different periodontal clinical conditions. Anaerobe. 2018;52:50–58. doi: 10.1016/j.anaerobe.2018.05.015 29857043

[47] Yang NY, Zhang Q, Li JL, et al. Progression of periodontal inflammation in adolescents is associated with increased number of porphyromonas gingivalis, Prevotella intermedia, tannerella forsythensis, and fusobacterium nucleatum. Int J Paediatr Dent. 2014;24(3):226–233. doi: 10.1111/ipd.12065 24025042

[48] Zheng X, Liu R, Zhou C, et al. ANGPTL4-mediated promotion of glycolysis facilitates the colonization of fusobacterium nucleatum in colorectal cancer. Cancer Res. 2021;81(24):6157–6170. doi: 10.1158/0008-5472.CAN-21-2273 34645607 PMC9397643

[49] Hashemi Goradel N, Heidarzadeh S, Jahangiri S, et al. Fusobacterium nucleatum and colorectal cancer: a mechanistic overview. J Cell Physiol. 2019;234(3):2337–2344. doi: 10.1002/jcp.27250 30191984

[50] Krisanaprakornkit S, Kimball JR, Weinberg A, et al. Inducible expression of human β-defensin 2 by fusobacterium nucleatum in oral epithelial cells: multiple signaling pathways and role of commensal bacteria in innate immunity and the epithelial barrier. Infect Immun. 2000;68(5):2907–2915. doi: 10.1128/IAI.68.5.2907-2915.2000 10768988 PMC97503

[51] Ahn SH, Chun S, Park C, et al. Transcriptome profiling analysis of senescent gingival fibroblasts in response to Fusobacterium nucleatum infection. PLoS One. 2017;12(11):e0188755. doi: 10.1371/journal.pone.0188755 29190775 PMC5708803

[52] Bhattacharyya S, Ghosh SK, Shokeen B, et al. FAD-I, a fusobacterium nucleatum Cell wall-associated diacylated lipoprotein that mediates human beta defensin 2 induction through toll-like receptor-1/2 (TLR-1/2) and TLR-2/6. Infect Immun. 2016;84(5):1446–1456. doi: 10.1128/IAI.01311-15 26930710 PMC4862701

[53] de Block T, González N, Abdellati S, et al. Successful intra- but not inter-species recombination of msr(D) in Neisseria subflava. Front Microbiol. 2022;13:855482. doi: 10.3389/fmicb.2022.855482 35432273 PMC9007320

[54] Liu B, Faller LL, Klitgord N, et al. Deep sequencing of the oral microbiome reveals signatures of periodontal disease. PLOS ONE. 2012;7(6):e37919. doi: 10.1371/journal.pone.0037919 22675498 PMC3366996

[55] Babikow E, Ghaltakhchyan N, Livingston T, et al. Longitudinal microbiome changes in supragingival biofilm transcriptomes induced by orthodontics. JDR Clin Transl Res. 2023;9(3):265–276. doi: 10.1177/23800844231199393 PMC11184915 37876206

[56] Lenartova M, Tesinska B, Janatova T, et al. The oral microbiome in periodontal health. Front Cell Infect Microbiol. 2021;11:629723. doi: 10.3389/fcimb.2021.629723 33828997 PMC8019927

[57] Chen M, Zhang C, Zhang X, et al. Meningococcal Quinolone Resistance Originated from Several Commensal Neisseria Species. Antimicrob Agents Chemother. 2020;64(2):64. doi: 10.1128/AAC.01494-19 PMC6985708 31740556

[58] Perera M, Al-Hebshi NN, Perera I, et al. Inflammatory bacteriome and oral squamous Cell carcinoma. J Dent Res. 2018;97(6):725–732. doi: 10.1177/0022034518767118 29630846

[59] Vieira AR, Hiller NL, Powell E, et al. Profiling microorganisms in whole saliva of children with and without dental caries. Clin Exp Dent Res. 2019;5(4):438–446. doi: 10.1002/cre2.206 31452955 PMC6704248

[60] Dashper SG, Mitchell HL, Ka LC, et al. Temporal development of the oral microbiome and prediction of early childhood caries. Sci Rep. 2019;9(1):19732. doi: 10.1038/s41598-019-56233-0 31874981 PMC6930300

[61] Uğar-Cankal D, Ozmeric N. A multifaceted molecule, nitric oxide in oral and periodontal diseases. Clin Chim Acta. 2006;366(1–2):90–100. doi: 10.1016/j.cca.2005.10.018 16387291

[62] Alamri MM, Williams B, Le Guennec A, et al. Metabolomics analysis in saliva from periodontally healthy, gingivitis and periodontitis patients. J Periodontal Res. 2023;58(6):1272–1280. doi: 10.1111/jre.13183 37787434

[63] Matejka M, Partyka L, Ulm C, et al. Nitric oxide synthesis is increased in periodontal disease. J Periodontal Res. 1998;33(8):517–518. doi: 10.1111/j.1600-0765.1998.tb02352.x 9879526

[64] Curtis MA, Diaz PI, Van Dyke TE, et al. The role of the microbiota in periodontal disease. Periodontol. 2020;83(1):14–25. doi: 10.1111/prd.12296 32385883

[65] Abusleme L, Hoare A, Hong BY, et al. Microbial signatures of health, gingivitis, and periodontitis. Periodontol. 2021;86(1):57–78. doi: 10.1111/prd.12362 33690899

[66] Sedghi L, DiMassa V, Harrington A, et al. The oral microbiome: role of key organisms and complex networks in oral health and disease. Periodontol. 2021;87(1):107–131. doi: 10.1111/prd.12393 PMC8457218 34463991

[67] Barbour A, Elebyary O, Fine N, et al. Metabolites of the oral microbiome: important mediators of multikingdom interactions. FEMS Microbiol Rev. 2022;46(1). doi: 10.1093/femsre/fuab039 34227664

[68] Hendrickson EL, Wang T, Beck DA, et al. Proteomics of Fusobacterium nucleatum within a model developing oral microbial community. Microbiologyopen. 2014;3(5):729–751. doi: 10.1002/mbo3.204 25155235 PMC4234264

[69] Karacaoglu F, Gazioglu C, Akkaya S, et al. Are the effects of fixed orthodontic treatment on gingival health similar in adolescents and young adults. J Biomed Sci. 2017;6(01):6. doi: 10.4172/2254-609X.100049

[70] Wojcicki CJ, Harper DS, Robinson PJ. Differences in periodontal disease-associated microorganisms of subgingival plaque in prepubertal, pubertal and postpubertal children. J Periodontol. 1987;58(4):219–223. doi: 10.1902/jop.1987.58.4.219 3473218

[71] Markou E, Eleana B, Lazaros T, et al. The influence of sex steroid hormones on gingiva of women. Open Dent J. 2009;3(1):114–119. doi: 10.2174/1874210600903010114 19812718 PMC2758498

