# Supplementary material for: Cross-omics analysis reveals microbe–metabolism interactions characteristic of gingival enlargement associated with fixed orthodontic in adolescents
Source: J Oral Microbiol. 2025 Jun 4;17(1):2513739. doi: 10.1080/20002297.2025.2513739 (PMC12138939; doi:10.1080/20002297.2025.2513739)
Supplement: Supplementary figures and methods.pdf [file ZJOM_A_2513739_SM3145.pdf]

Supplementary methods

Sample collection

Saliva samples were collected according to Alamri's method[1], with one difference: two samples of saliva were collected from each subject in sterile cryopreservation tubes for metagenomics and metabolomic analyses, and immediately sent to liquid nitrogen (LN) tanks at -196 ℃ for storage. All samples were sent to the BGI for sequencing analysis, and were transported by cold chain.

Shotgun metagenome sequencing and data analysis

Extraction of Microbiome DNA

Prepare 5 96-well deep plates，Add 600 μL Buffer with magnetic beads +20 μL Proteinase K+5 μL RNase A, 700 μL Wash 1, 700 μL Wash 2, 700 μL Wash 3, 100 μL Elution Buffer. Transfer 100-200 mg sample to the centrifuge tube with grinding beads. Add 1 mL Buffer ATL/PVP-10, grinding the sample with the grinding machine and incubate at 65℃ for 20 min. Centrifuge at 14000×g for 5 minutes. Transfer the supernatant to a new tube. Add 0.6 mL Buffer PCI，Mix thoroughly by vortex 15s. Centrifuge at 18213×g for 10 minutes. Transfer the supernatant to deep well plate with magnetic beads binding solution. Transfer the deep well plate to the proper place of the machine. Start the corresponding program in Kingfisher. Transfer the DNA to 1.5 mL centrifuge tube.

Metagenome Library Preparation (DNBSEQ)

Samples underwent quality control based on sample type and product requirements. The genomic DNA was randomly fragmented, and the fragmented DNA was selected to achieve a desired average size. End-repair and 3' adenylation were performed on the fragments, followed by ligation of adaptors to the 3' adenylated ends. PCR reaction was set up to amplify the product. The library was subjected to quality control using an appropriate protocol based on the product requirements. To generate single-stranded PCR products, denaturation was carried out. The reaction system and program were then configured for circularization. This resulted in the production of single-stranded cyclized products, while uncyclized linear DNA molecules were digested. For sequencing, the single-stranded circular DNA molecules were replicated through rolling cycle amplification, generating DNA nanoballs (DNBs) that contained multiple copies of DNA. High-quality DNBs were loaded onto patterned nanoarrays using the high-intensity DNA nanochip technique.

Sequencing was performed using combinatorial Probe-Anchor Synthesis (cPAS).

Metagenomic analysis

All the raw data were trimmed by SOAPnuke v.2.2.1 [2]. The trimmed reads were mapped to the host genome using SOAP2 [3] software, which identified and removed host-originating reads. High-quality reads were assembled de novo using MEGAHIT [4] software. Assembled contigs with length less than 300 bp were discarded in the following analysis. Genes were predicted across contigs by using MetaGeneMark [5]. Redundant genes were eliminated using CD-HIT [6] with the identity and coverage cutoff 95%, 90%, respectively. In order to construct gene abundance matrix, Salmon [7] software is used quantify gene abundance and create a matrix. The TPM value obtained is the normalised gene abundance value. the TPM quantification formula is shown below.

$$TPM_i = \frac{X_i}{\tilde{l}_i} \cdot \left(\frac{1}{\sum_j \frac{X_j}{\tilde{l}_j}} \right) \cdot 10^6$$

To generate the annotation information, the protein sequences of genes were aligned against the functional database (such as BacMet, CARD, KEGG, EggNOG, COG, Swiss-Prot, CAZy, etc.) using DIAMOND[8] with an E value cutoff of 1e−5. Based on the Kraken[9] LCA algorithm, the taxonomic annotation was assigned. To generate the taxonomic and functional abundance profiles, the Bracken(https://github.com/jenniferlu717/Bracken) software is used with the default setting.

Wilcoxon's rank sum test was used to identify characteristics (genera, phyla, and KOs) with substantially different abundances across groups based on the abundance profiles[10]. KEGG pathways that were differentially enriched were identified using reporter scores[11]. The significance threshold was set at an absolute value of 1.65 or greater for the reporter score. The Shannon index, chao1 index, and Simpson index were used with the R program to quantify alpha diversity based on relative abundance profiles at the gene, genus, and KO levels. Beta diversity was determined using the Euclidean distance, the Bray-Curtis distance[12], and the Jensen-Shannon Divergence distance[13]. The Wilcoxon rank test and Kruskal-Wallis H test were analyzed statistically using the R project.

Untargeted metabolomics sequencing and data processing

Metabolite extraction was carried out using a previously described method[14], and the prepared supernatant was used for LC-MS analysis using a Waters Iclass UPLC (Waters, USA) in conjunction with an Exploris 480 high-resolution mass spectrometer (Thermo Fisher Scientific, USA) for metabolite separation and detection. Metabolite data were gathered using both positive and negative ion modalities to increase metabolite coverage and accuracy.

To extract and identify metabolite peaks, raw mass spectrometry data was loaded into Compound Discoverer 3.3 software. The mass spectrometry data was analyzed using databases such as BMDB, mzCloud, and ChemSpider, which produced a data matrix combining metabolite peak areas and identification results. The results exported by Compound Discoverer were then loaded into metaX for data preparation. The data preprocessing steps involved the following procedures: normalizing of the data using the Probabilistic Quotient Normalization (PQN) method [15] to obtain relative peak areas; correcting of batch effects using the Quality Control-based Robust LOESS Signal Correction (QC-RLSC) method; and removing compounds with a coefficient of variation (CV) greater than 30% in QC samples. Folowing log2 transformation and Pareto scaling of original data, principal component analysis (PCA) was used to evaluate trends in sample separation and identify potential outliers. Metabolite classification and functional annotation were carried out on databases such as HMDB and KEGG. The Variable Important for the Projection (VIP) values were computed in order to pick metabolite biomarkers, and differences between groups were assessed using the Partial Least Squares Discriminant Analysis (PLS-DA) approach. Using Pareto scaling and 7-fold cross-validation, PLS-DA models were created for group comparison analysis.

Figure S1

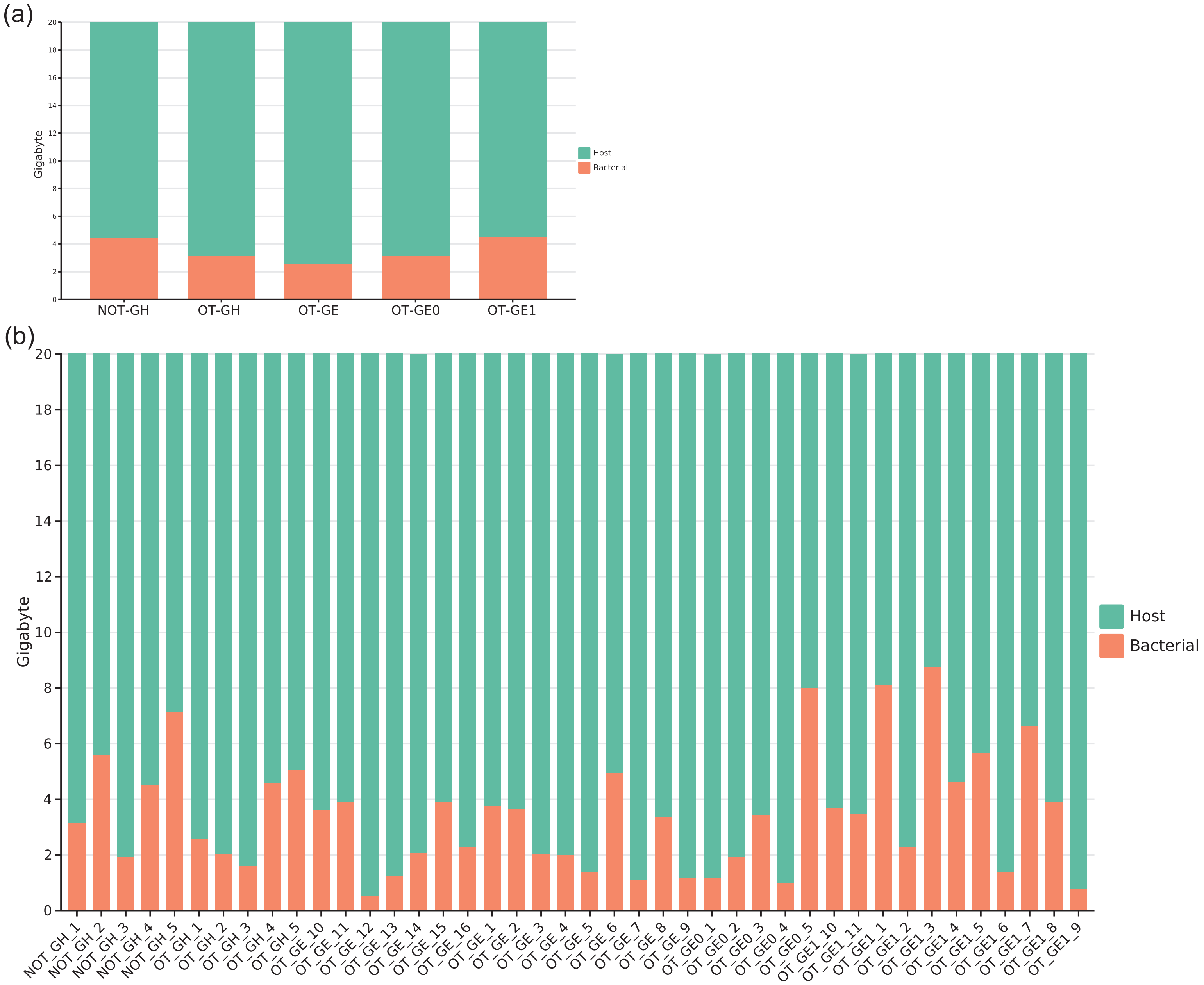

Supplementary figure S1(a) The averaged ratio of bacterial reads to the host reads in the control and case group. (b) The amounts of bacterial reads to the host reads in each sample after multiple rounds of sequencing. NOT-GH, no orthodontic treatment group of periodontal health; OT-GH, Orthodontic treatment group of periodontal health; OT-GE, Group of orthodontic treatment-induced gingival enlargement; OT-GE0, Group of no gingival enlargement after periodontal nonsurgical treatment; OT-GE1, Group of gingival enlargement after periodontal nonsurgical treatment.

Figure S2

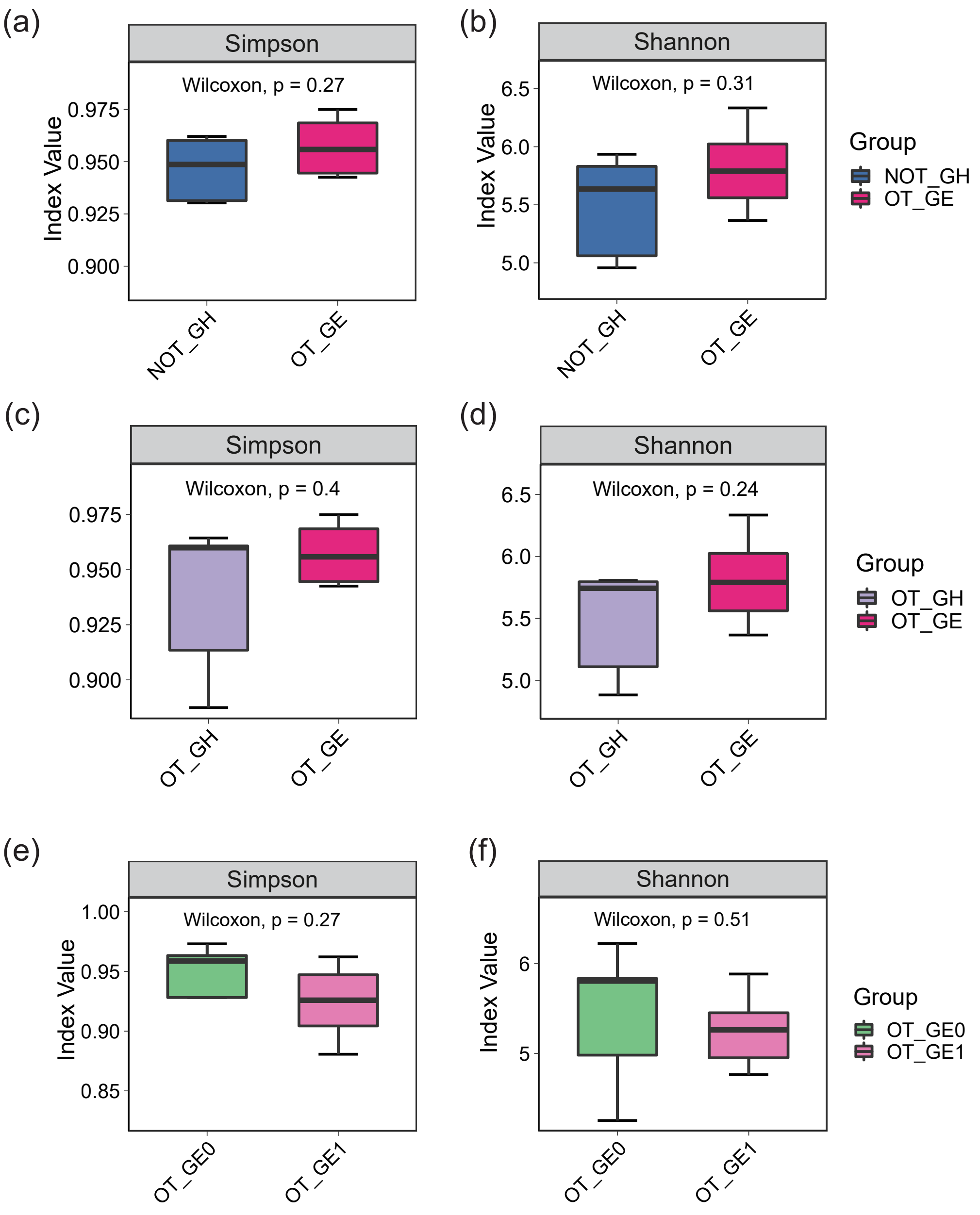

Supplementary figure S2 The richness and diversity of the microbiota in the case and control groups were grouped according to NOT-GH vs. OT-GE (a, b), OT-GH vs. OT-GE (c, d) and OT-GE0 vs. OT-GE1 (e, f), respectively.NOT-GH, no orthodontic treatment group of periodontal health; OT-GH, Orthodontic treatment group of periodontal health; OT-GE, Group of orthodontic treatment-induced gingival enlargement; OT-GE0, Group of no gingival enlargement after periodontal nonsurgical treatment; OT-GE1, Group of gingival enlargement after periodontal nonsurgical treatment.

Figure S3

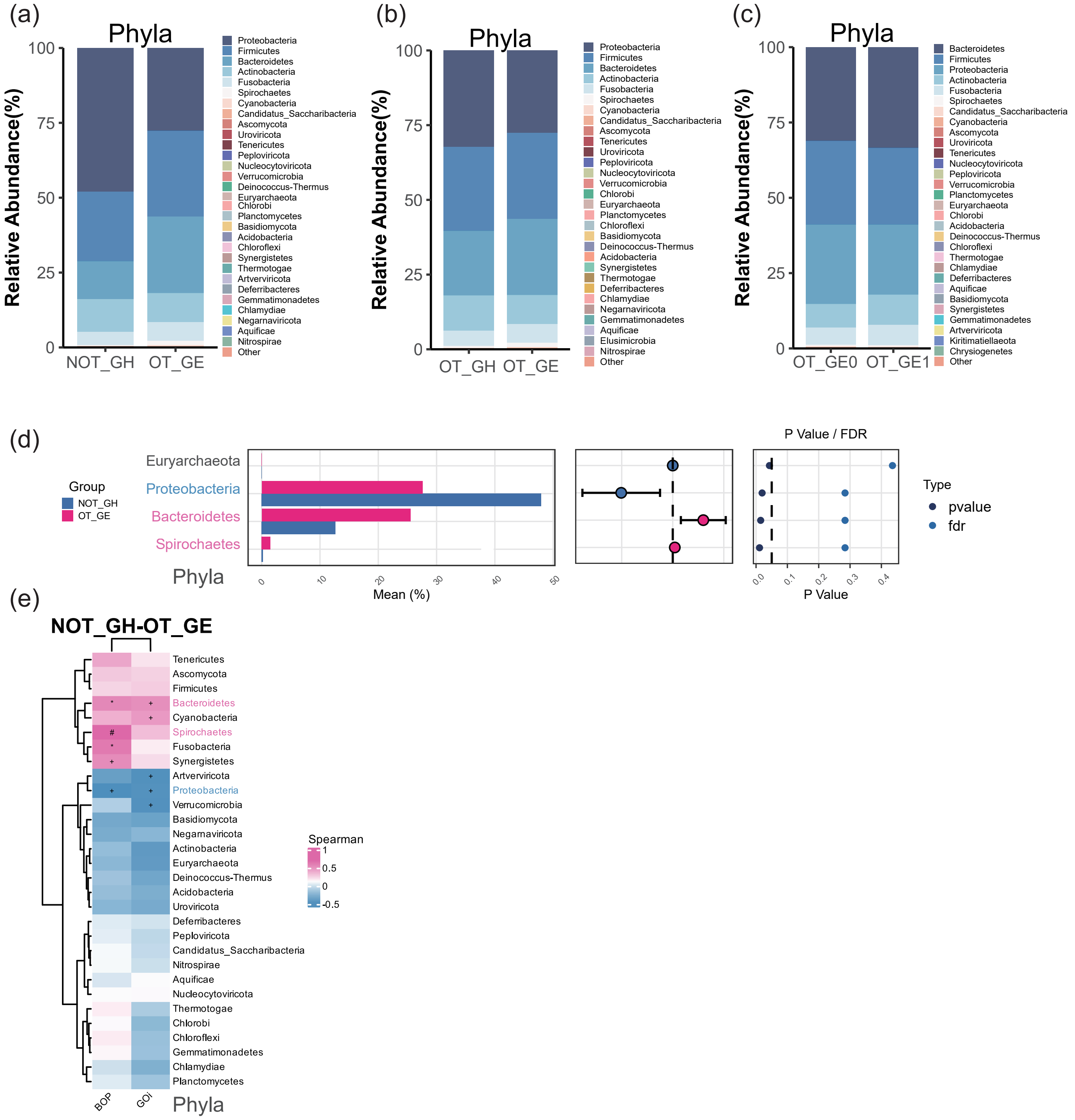

Supplementary figure S3 Characterisation of oral microbial species at the phylum level associated with orthodontic treatment-induced gingival enlargement. (a-c) Relative abundance of NOT-GH & OT-GE, OT-GH & OT-GE, and OT-GE0 & OT- GE1 at the bacterial phylum level in the oral microbiota (Supplementary Tables 1-3). (d) Significant differences between microorganisms were identified by phylum-level System Theoretic Accident Model and Processes (STAMP) analysis (Supplementary Tables 10-12 show that only NOT-GH & OT-GE had significantly different phyla). (e) Heat map of Spearman rank correlation coefficients between BOP, GOi, and phylum-level differential species ($P < 0.05$ marked as ‘+’; $P < 0.01$ marked as ‘*’; $P < 0.001$ marked as ‘# ’; $P > 0.05$ not labelled, Supplementary Tables 19-21 show only NOT-GH & OT-GE are statistically different).

Figure S4

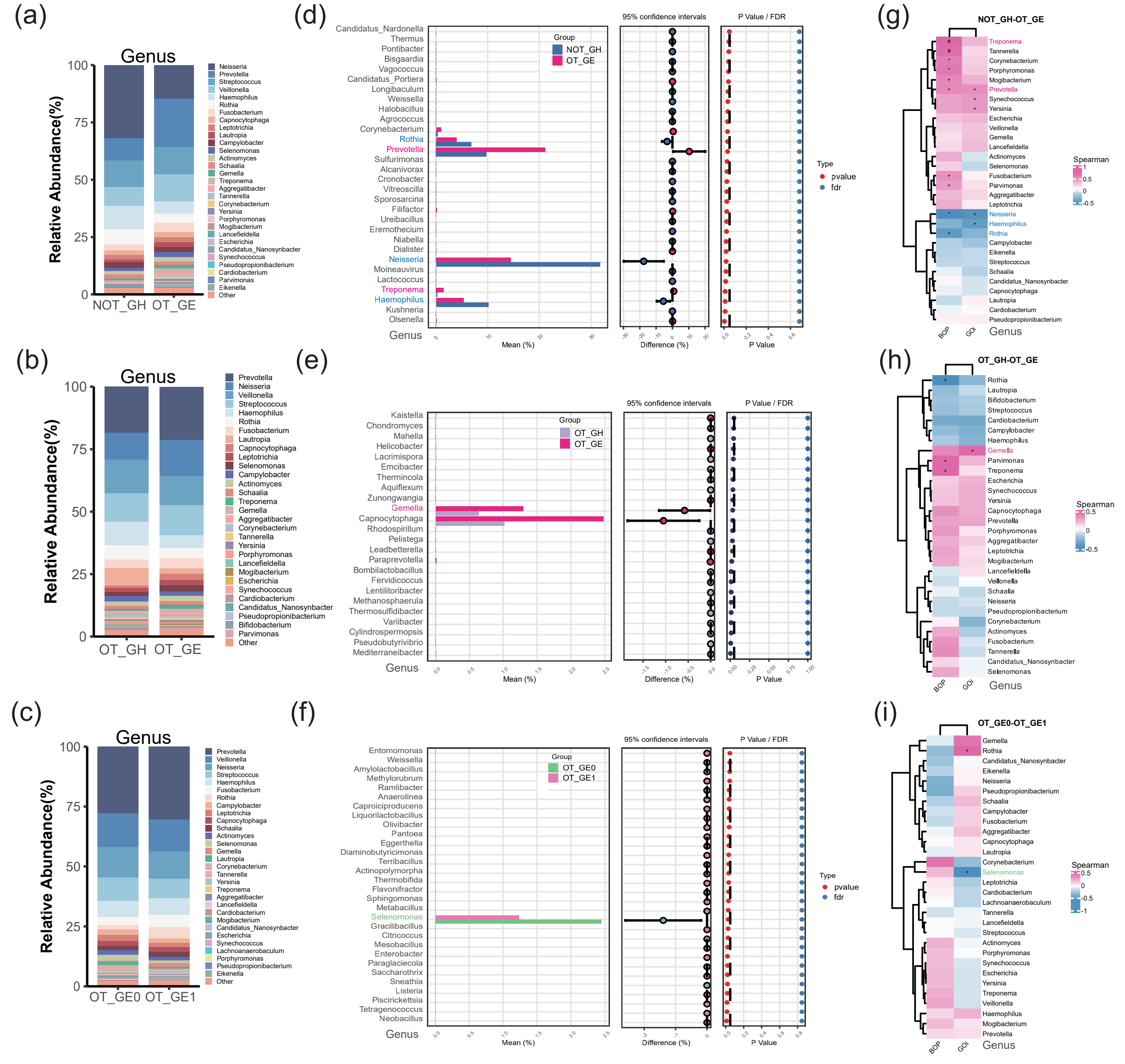

Supplementary figure S3 Characterizations of genus-level oral microbial species associated with orthodontic treatment-induced gingival enlargement. (a-c) Relative abundance of NOT-GH & OT-GE, OT-GH & OT-GE, and OT-GE0 & OT- GE1 at the bacterial genus level in the oral microbiota (Supplementary Tables 4-6). (d-f) Significant differences between microorganisms were identified by genus level System-Theoretic Accident Model and Processes (STAMP) analysis (Supplementary Tables 13-15). Heat map of Spearman rank correlation coefficients between BOP, GOi, and genus-level differential species (P < 0.05 marked as '+'; P < 0.01 marked as '*'; P < 0.001 marked as '# '; P > 0.05 not labelled, Supplementary Tables 22-24).

Figure S5

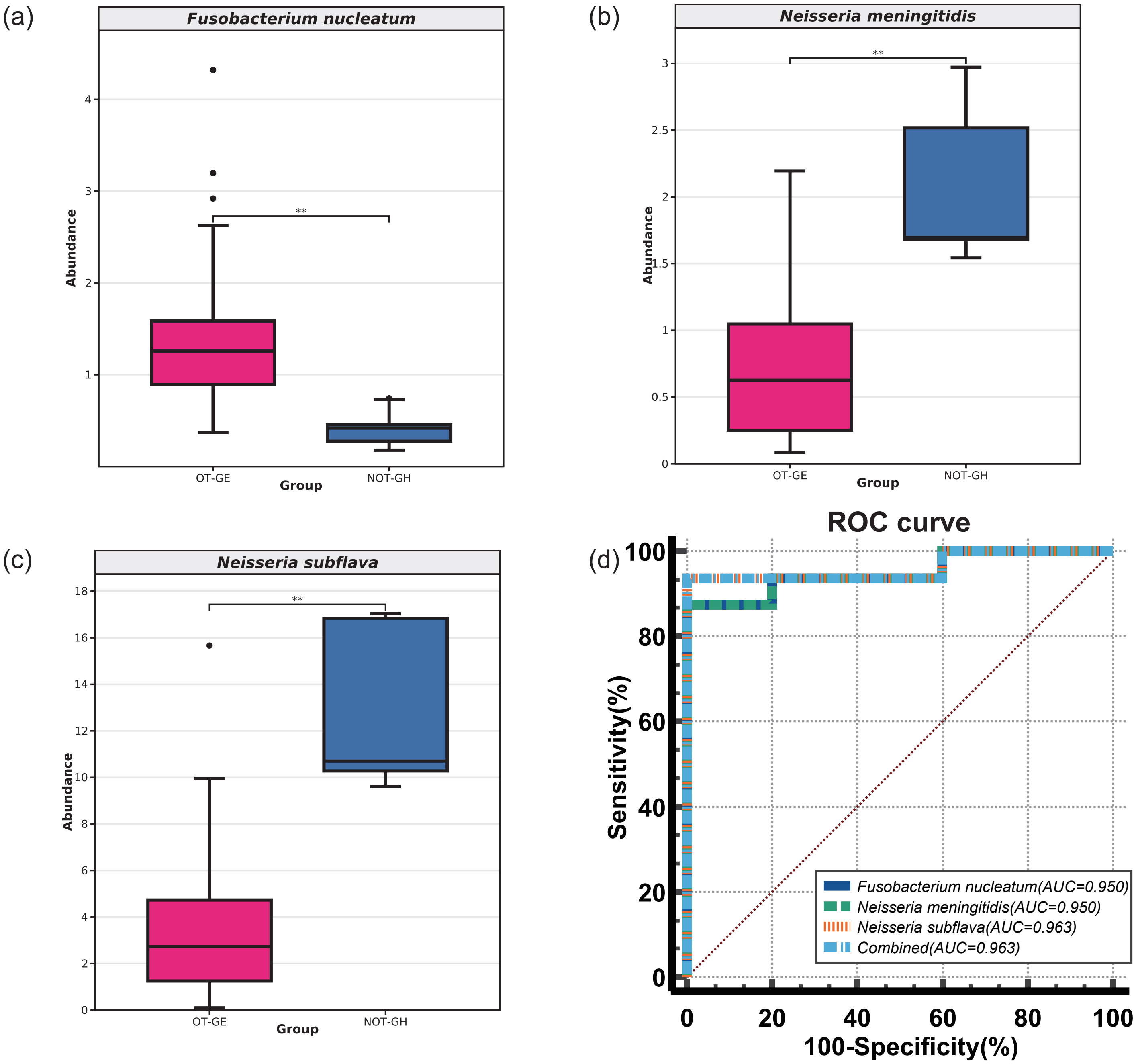

Supplementary Figure S5 Key strains distinguish between NOT-GH and OT-GE groups. (a-c) Box plots of the groupings based on the distribution of the sample species. the Wilcoxon test was used for statistical analysis, ** represents p<0.01; * represents p<0.05.(d) ROC curves depicting the change in species classification as the species or the combination as a measure of biomarker ability. The horizontal coordinate is 100-specificity (%) and the vertical coordinate is sensitivity (%). The area under the line is the AUC value. A larger AUC value indicates that the metabolite is more suitable as a biomarker.NOT-GH, no orthodontic treatment group of periodontal health; OT-GE, Group of orthodontic treatment-induced gingival enlargement.

Figure S6

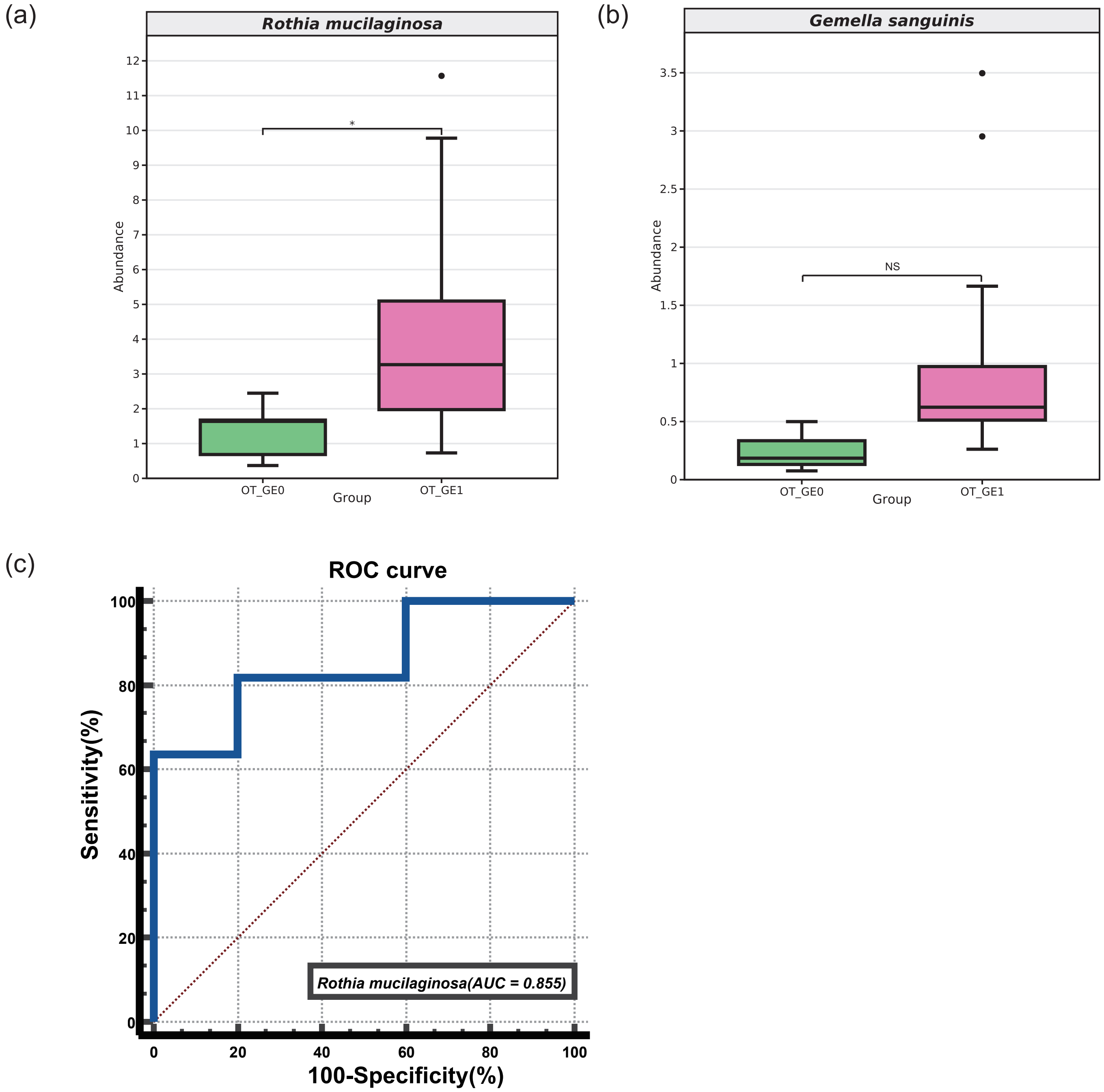

Supplementary Figure S6 Key strains distinguish between OT-GE0 and OT-GE1 groups. (a-c) Box plots of the groupings based on the distribution of the sample species. the Wilcoxon test was used for statistical analysis, ** represents p<0.01; * represents p<0.05.(d) ROC curves depicting the change in species classification as the species or the combination as a measure of biomarker ability. The horizontal coordinate is 100-specificity (%) and the vertical coordinate is sensitivity (%). The area under the line is the AUC value. A larger AUC value indicates that the metabolite is more suitable as a biomarker.OT-GE0, Group of no gingival enlargement after periodontal nonsurgical treatment; OT-GE1, Group of gingival enlargement after periodontal nonsurgical treatment.

Figure S7

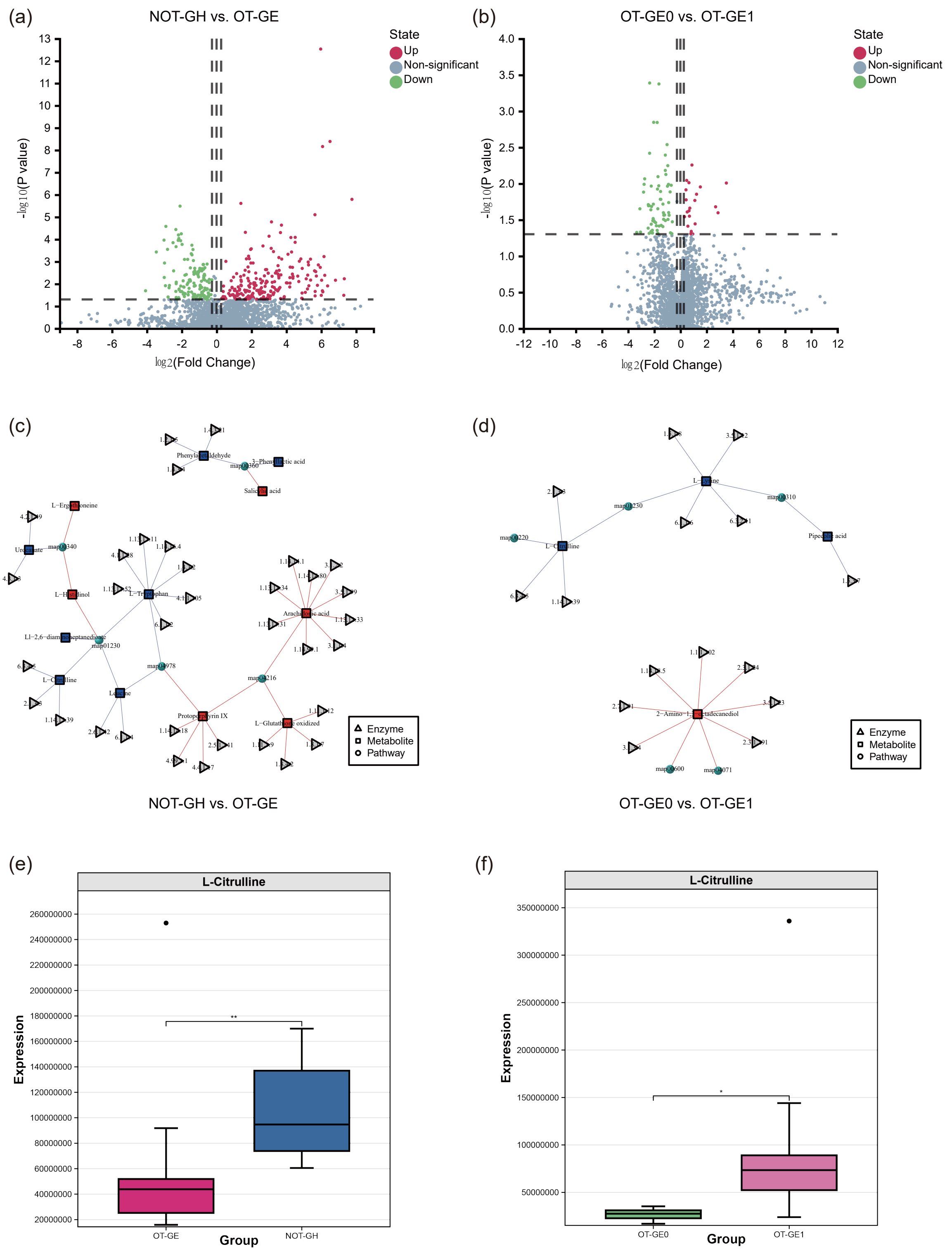

Supplementary figure S7 Key differential metabolite content and functional enrichment analyses of the NOT-GH vs. OT-GE comparison group and the OT-GE0 vs. OT-GE1 comparison group. (a,b) Differential metabolite volcano plots. Horizontal coordinates are log2-transformed Fold Change and vertical coordinates are -log10-transformed q-value (pvalue). Down-regulated significantly different metabolites are shown in green, up-regulated significantly different metabolites in red, and non-significant metabolites in grey. (c,d) Network diagram of differential metabolite enrichment analysis. Metabolic pathway enrichment analysis of differential metabolites was performed based on KEGG database, and metabolic pathways with pvalue < 0.05 were defined as metabolic pathways significantly enriched for differential metabolites, and the top 5 metabolic pathways with the smallest pvalue (less than 5, all data were used) were plotted. Dots represent a metabolic pathway, and triangles and squares connected to the dots indicate enzymes or metabolites in that pathway. Squares represent differential metabolites, with red indicating up-regulation and blue indicating down-regulation. Grey triangles represent information on the regulatory enzyme associated with the metabolite, which has been annotated by species. (e,f) L-Citrulline grouped box plot content analysis. the Wilcoxon test was used for statistical analysis, ** represents p<0.01; * represents p<0.05. NOT-GH, no orthodontic treatment group of periodontal health; OT-GE, Group of orthodontic treatment-induced gingival enlargement; OT-GE0, Group of no gingival enlargement after periodontal nonsurgical treatment; OT-GE1, Group of gingival enlargement after periodontal nonsurgical treatment.

Figure S8

(a)

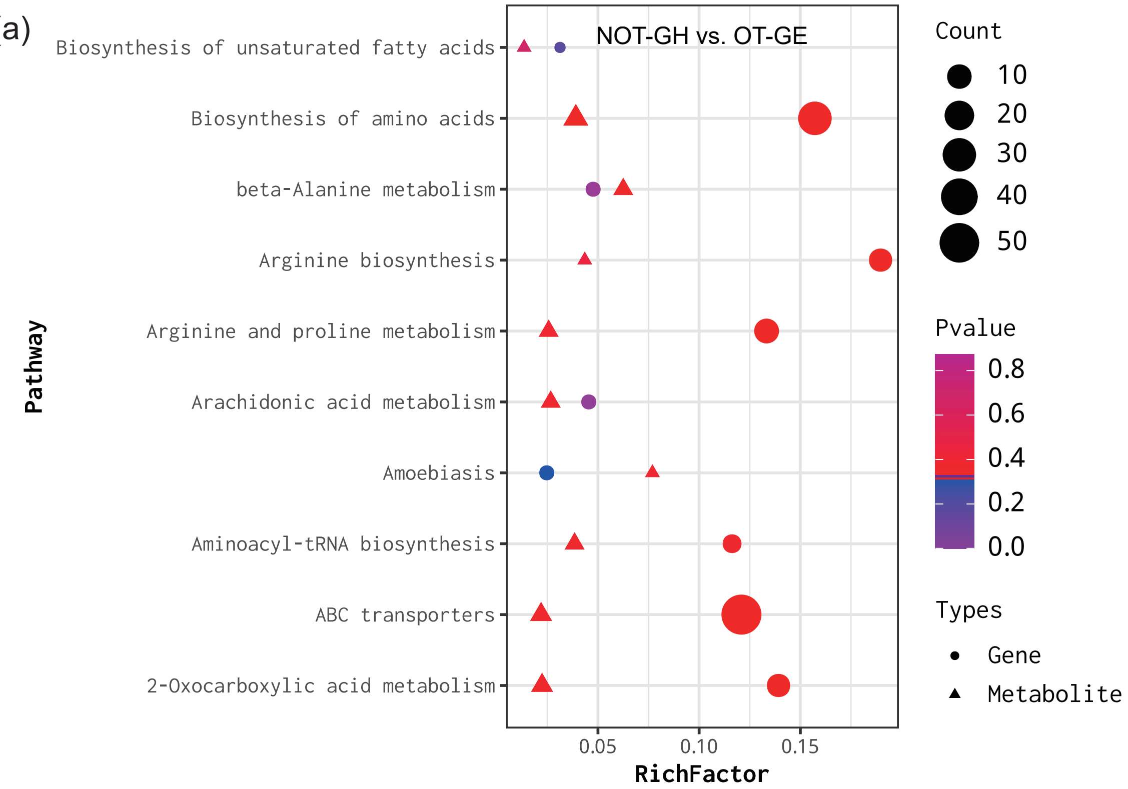

(b)

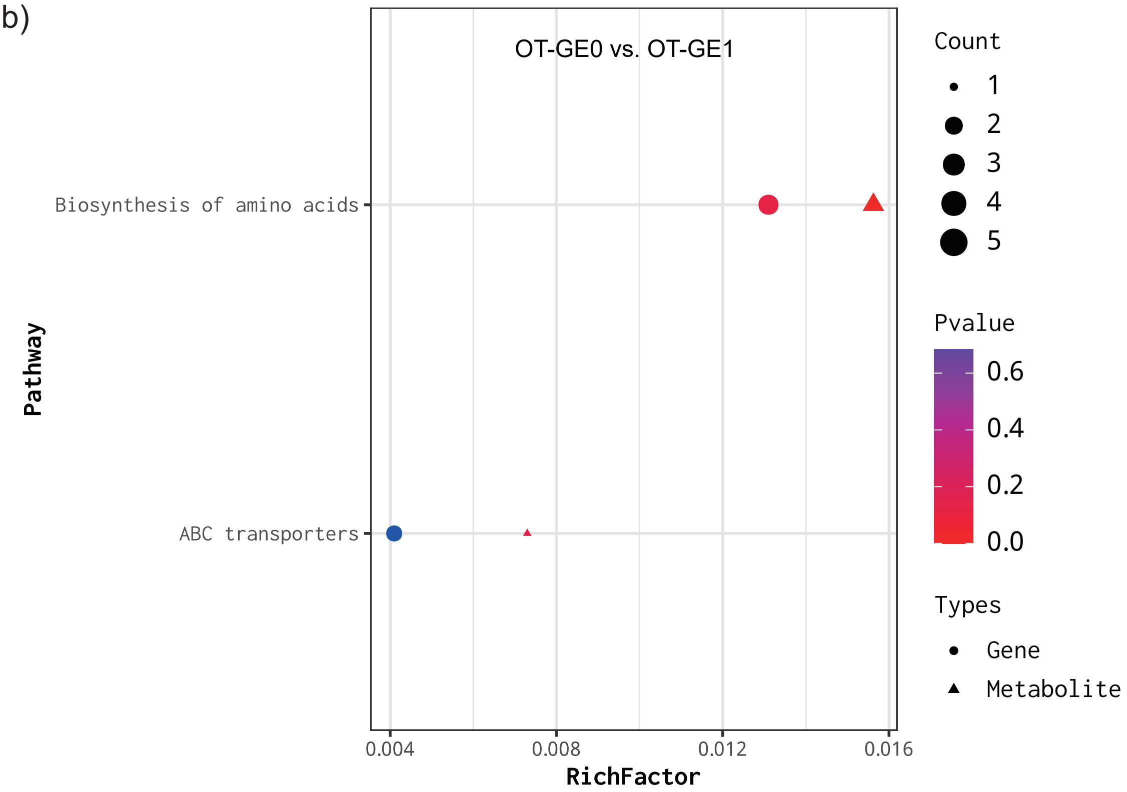

Supplementary figure S8 Functional gene pathway enrichment analysis. By performing pathway enrichment analysis on functional genes and metabolites, the degree of enrichment of functional genes and metabolites in each pathway is calculated. The bubble diagrams of Pathways with significant enrichment of differential genes and differential metabolites are shown in Figures a and b. The X-axis RichFactor, the larger the value, the larger the proportion of differential genes and differential metabolites annotated to the Pathway. The triangles represent functional gene pathways, the circles represent metabolic pathways, the size of the graph represents the number of differential genes and differential metabolites annotated to the Pathway, and the colour of the graph indicates the significance of the pathway.NOT-GH, no orthodontic treatment group of periodontal health; OT-GE, Group of orthodontic treatment-induced gingival enlargement; OT-GE0, Group of no gingival enlargement after periodontal nonsurgical treatment; OT-GE1, Group of gingival enlargement after periodontal nonsurgical treatment.

[1] M.M. Alamri, B. Williams, A. Le Guennec, et al., Metabolomics analysis in saliva from periodontally healthy, gingivitis and periodontitis patients,[J] Journal of periodontal research 58(6) (2023) 1272-1280.
[2] Y. Chen, Y. Chen, C. Shi, et al., SOAPnuke: a MapReduce acceleration-supported software for integrated quality control and preprocessing of high-throughput sequencing data,[J] GigaScience 7(1) (2018) 1-6.
[3] R. Li, C. Yu, Y. Li, et al., SOAP2: an improved ultrafast tool for short read alignment,[J] Bioinformatics (Oxford, England) 25(15) (2009) 1966-7.
[4] D. Li, C.M. Liu, R. Luo, et al., MEGAHIT: an ultra-fast single-node solution for large and complex metagenomics assembly via succinct de Bruijn graph,[J] Bioinformatics (Oxford, England) 31(10) (2015) 1674-6.
[5] W. Zhu, A. Lomsadze, M. Borodovsky, Ab initio gene identification in metagenomic sequences,[J] Nucleic acids research 38(12) (2010) e132.
[6] L. Fu, B. Niu, Z. Zhu, et al., CD-HIT: accelerated for clustering the next-generation sequencing data,[J] Bioinformatics (Oxford, England) 28(23) (2012) 3150-2.
[7] R. Patro, G. Duggal, M.I. Love, et al., Salmon provides fast and bias-aware quantification of transcript expression,[J] Nature methods 14(4) (2017) 417-419.
[8] B. Buchfink, C. Xie, D.H. Huson, Fast and sensitive protein alignment using DIAMOND,[J] Nature methods 12(1) (2015) 59-60.
[9] D.E. Wood, J. Lu, B. Langmead, Improved metagenomic analysis with Kraken 2,[J] Genome biology 20(1) (2019) 257.
[10] R.A. Matsouaka, A.B. Singhal, R.A. Betensky, An optimal Wilcoxon-Mann-Whitney test of mortality and a continuous outcome,[J] Statistical methods in medical research 27(8) (2018) 2384-2400.
[11] K.R. Patil, J. Nielsen, Uncovering transcriptional regulation of metabolism by using metabolic network topology,[J] Proceedings of the National Academy of Sciences of the United States of America 102(8) (2005) 2685-9.
[12] J.R. Bray, Curtis, J. T., et al., An Ordination of the Upland Forest Communities of Southern Wisconsin,[J] 27 (1957) 325-349.
[13] A.P. Majtey, P.W. Lamberti, D.P.J.P.R.A. Prato, Jensen-Shannon divergence as a measure of distinguishability between mixed quantum states,[J] 72 (2005) 052310.
[14] W.B. Dunn, D. Broadhurst, P. Begley, et al., Procedures for large-scale metabolic profiling of serum and plasma using gas chromatography and liquid chromatography coupled to mass spectrometry,[J] Nature protocols 6(7) (2011) 1060-83.
[15] R. Di Guida, J. Engel, J.W. Allwood, et al., Non-targeted UHPLC-MS metabolomic data processing methods: a comparative investigation of normalisation, missing value imputation, transformation and scaling,[J] Metabolomics : Official journal of the Metabolomic Society 12 (2016) 93.
